# LC–MS analysis of polar and highly polar organic pollutants in Barcelona urban groundwater using orthogonal LC separation modes

**DOI:** 10.1007/s11356-024-33471-y

**Published:** 2024-04-26

**Authors:** Francesc Labad, Sandra Pérez

**Affiliations:** Department of Environmental Chemistry, Institute of Environmental Assessment and Water Research (IDAEA), ONHEALTH, CSIC, Jordi Girona 18-26, 08034 Barcelona, Spain

**Keywords:** Pharmaceuticals, Industrial chemicals, VAE, LC-HRMS/MS Q Exactive Orbitrap

## Abstract

Groundwater samples may contain thousands of organic pollutants from infiltration of surface water, sewer leakages, and to a minor extent from public water supply network losses. Polar (0 < log *D* <  − 2.0) and very polar substances (log *D* <  − 2.0) have been largely beyond the scope of applied analytical methodologies in environmental monitoring because of challenges related to their extraction from the sample and subsequent chromatographic separation. In this study, we developed an analytical workflow for 96 pollutants covering a broad polarity range, including pharmaceuticals, industrial chemicals, and artificial sweeteners, potentially seeping through the soil in urban areas. The Besos aquifer located at the Northern-eastern edge of the city of Barcelona was chosen as a study area due to the deterioration of the quality of the aquifers over the past years and the proven presence of numerous pollutants. The methodology consisted of vacuum-assisted evaporation (VAE) followed by chromatographic separation of the sample on two columns with orthogonal retention mechanisms, namely, an HSS T3 column (modified C18) and a BEH amide column (HILIC). The analytes were detected by high-resolution mass spectrometry on a Q Exactive Orbitrap system in data-independent acquisition mode. Taking into consideration the retention as well as the peak shape, a Quality Score (QS) was assigned for each analyte to evaluate the quality of each chromatographic peak of each compound. While 67 compounds, including 19 polar and 48 moderately polar, were satisfactorily retained on an HSS T3, 29 compounds, including 14 highly polar, 14 polar, and one moderately polar, were analyzed in the BEH amide column. The optimized methodology was applied for the analysis of 89 out of 96 validated contaminants with satisfactory recoveries in samples collected from seven wells, providing low LODs (0.02 to 0.45 ng L^−1^) and LOQs (0.06 to 1.34 ng L^−1^). A number of highly polar and polar compounds not previously reported to occur in GW, including artificial sweeteners, pharmaceuticals, and industrial chemicals, were detected at concentrations as high as few μg L^−1^.

**Supplementary Information:**

The online version contains supplementary material available at 10.1007/s11356-024-33471-y.

## Introduction

In southern Europe, as a result of the limited availability of surface water and the growing demand for drinking water, alternative water resources such as groundwater (GW) need to be explored. Although GW can be thought to be protected from surface impacts by pollutant adsorption and degradation processes occurring during water infiltration (Arias-Estévez et al. 2008), there is a risk of pollution from seepage of surface water, sewer leakages, and to a minor extent from public water supply networks losses (Bester et al. 2008; Osenbrück et al. 2007; Schulze et al. 2018). When urban GW is located in a deltaic zone, like the Besos River north of the Barcelona metropolitan area, an additional threat is seawater intrusion (Jurado et al. 2012 and 2014; Tubau et al. 2014; López-Serna et al. 2013; Vázquez-Suñé et al. 2010). Therefore, GW is potentially polluted with a multitude of organic xenobiotics of diverse physico-chemical properties. Whereas many non-polar compounds can be retained in the soil, compounds of high polarity are more susceptible to reaching GW (Reemtsma et al. 2006; Schulze et al. 2019). The two major parameters quantitatively describing the polarity of an organic compound are the partition coefficient (log *P*) and the pH-dependent distribution coefficient (log *D*). They influence their mobility and ultimately GW vulnerability. Recent studies have classified compounds with log *D* 0–2 as polar and those with log *D* < 0 as very polar (Neumann and Schliebner 2019; Peter Arp and Hale 2019).

Several studies have demonstrated the presence of non-polar pollutants in GW (Grujić et al. 2009; Heberer et al. 2004; Loos et al. 2010; Wolf et al. 2012) across Europe. In two studies from German groundwater, assessing the presence of six 6 PhACs (Heberer et al. 2004) and 22 personal care products (Wolf et al. 2012), respectively, the levels ranged from non-detected to 4.2 µg L^−1^. A Serbian study demonstrated the presence of four PhACs (azithromycin, carbamazepine, paracetamol, and trimethoprim) (Grujić et al. 2009). A pan-European study involving 23 countries investigated the presence of 59 contaminants in 164 GW samples, with the most frequently detected compounds being DEET, caffeine, PFOA, and atrazine (Loos et al. 2010). Most of the studies were focused on the occurrence of non-polar and medium-polar substances, whereas polar and very polar pollutants were largely outside the scope of the analytical methodologies applied because of challenges related to their extraction from the sample and subsequent chromatographic separation. The few studies related to polar substances in GW relate to pesticides (Köck-Schulmeyer et al. 2014), some industrial chemicals (Heberer et al. 1998; Schorr et al. 2023), and one study treating with surface water and effluent and influent wastewater, demonstrating the presence up to 27 polar chemicals (log *D* − 6 to 1), combining pharmaceuticals and industrial compounds (Mechelke et al. 2019).

As far as sample enrichment is concerned, solid-phase extraction (SPE) is the most common method for the preconcentration of organic pollutants in aqueous samples (Huntscha et al. 2012; López-Serna et al. 2011; Richardson et al. 2017; Ruff et al. 2015). Nevertheless, most commercially available SPE cartridges, such as the popular hydrophilic-lipophilic balance (HLB) sorbent (Dias and Poole 2002), have been demonstrated to poorly retain polar to very-polar compounds because they were designed for extracting non to medium-polar compounds. Conversely, graphitized carbon black (GCB) with its high retention capability for polar compounds exhibits severe problems in their desorption (Schulze et al. 2019). To address selectivity issues related to the use of single sorbent, multi-sorbent SPE (msSPE) has been proposed; it consists of an SPE cartridge filled with a (layered) combination of sorbents such as HLB and ion exchange resins (di Corcia et al. 1993; Fagnani et al. 2022; Gilart et al. 2014; Köke et al. 2018; Osorio et al. 2016). For the sake of simplicity and ease of use, alternative preconcentration methods with reduced risk of analyte losses were developed including enrichment by evaporative techniques (Angeles and Aga 2020; Köke et al. 2018; Mechelke et al. 2019; Schulze et al. 2020). Vacuum-assisted evaporation (VAE) has several advantages over msSPE, such as time-saving, solvent-free sample extraction, cost-saving, and minimal sample handling.

Besides the critical sample enrichment step, poor analyte retention is commonly observed when using typical reversed-phase liquid chromatographic (RPLC) columns, which have been successfully employed in the past for multi-class organic contaminant separations (Ruff et al. 2015). Therefore, novel retention modes have been explored in recent years to enhance the retention of polar organic compounds, such as hydrophilic interaction liquid chromatography (HILIC) (Reemtsma et al. 2016; Tang et al. 2016; van Nuijs et al. 2011; Montes et al. 2019). To the best of the author’s knowledge, no HILIC-based method for the analysis of highly polar substances in urban groundwaters has been previously reported in the literature.

Here, we propose an analytical procedure using two orthogonal LC columns for the quantitative determination of groundwater pollutants with different polarities; the normal phase with a polar stationary phase (used for the retention of polar to highly-polar substances) and the reversed phase, with opposite retention principle, retaining non-polar to medium-polar substances. It allowed for sensitive measurement of the target analytes in urban GW samples from a polluted area.

## Materials and methods

### Chemicals, reagents, and solutions

The analytes included in this work were selected based on their environmental occurrence and inclusion in watch lists (European Parliament and of the Council 2013; Peter Arp and Hale 2019). The list included 96 compounds with a wide range of polarities (from log *D*-6 to 2) of which were classified as highly polar (log *D* <  − 2), polar (0 > log *D* >  − 2), and moderately polar compounds (0 < log *D* < 2). Analytical reference standards used for method development were of high purity (Sigma Aldrich (St. Louis, MO, U.S.). Isotope-labeled compounds used as surrogates were purchased from Cerilliant (Sigma Aldrich), Santa Cruz Biotechnology (Dallas, TX, USA), or Toronto Research Chemicals (Toronto, ON, Canada). Chemical structures and relevant properties of the target compounds are compiled in Table S1.

For stock solution and sample preparation, LC–MS grade acetonitrile (ACN, ≥ 99.9%), methanol (MeOH, ≥ 99.9%), ethyl acetate (EtOAc, ≥ 99.9%), dimethyl sulfoxide (DMSO, ≥ 99.9%), and HPLC water were purchased from Merck (Darmstadt, Germany). Formic acid (FA, ≥ 96.0%, ACS grade), ammonium acetate, and ammonia solution (29.0%, ACS grade) were supplied by Sigma Aldrich. LC/MS Optima™ Grade ACN, water, and ammonium fluoride for the preparation of the mobile phase were purchased from Fisher Chemical (Fisher Scientific SL, Madrid, Spain). Individual analyte stock solutions were prepared at a concentration of 1000 μg mL^−1^ in either MeOH, ACN, DMSO, or HPLC water, depending on the solubility of each compound, and stored at − 20 °C. Mixed working solutions of the analytes and the isotopically labeled compounds were prepared in MeOH at 2 μg mL^−1^ from stock solutions for method development, validation study, and calibration purposes.

### Sample collection

**Fig. 1:**
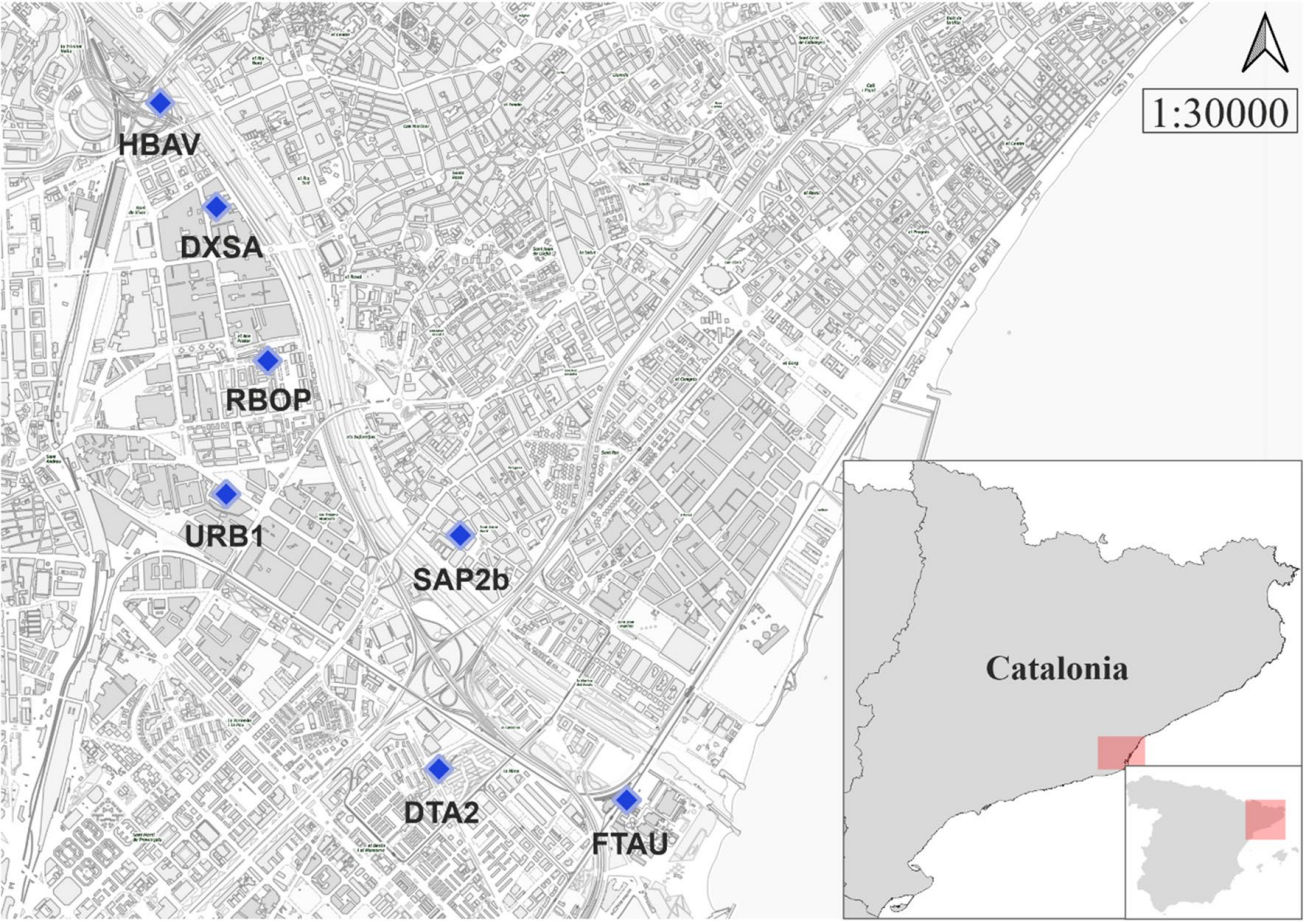
Location of the seven sampled GW wells in the vicinity of Besos River

The study area was located in the northeast of Spain, specifically, in the Besos River Delta, which includes the municipalities of Barcelona, Santa Coloma de Gramenet and Badalona, and the entire municipality of Sant Adria del Besos. In July 2020, 1-L groundwater samples from along the Besos River were collected from seven wells (Fig. 1, Table S6), in amber polyethylene terephthalate (PET) bottles, and stored at − 20 °C until analysis. Before collection, each well was pumped for 10 to 15 min to ensure a representative aliquot of the groundwater well. Sample parameters such as pH, electric conductivity, temperature, and hardness were measured in situ and are shown in Table S2. The water in the shallow aquifer is a mixture of recharged waters that come from different sources, such as river bank infiltration from the Besos River, infiltration of rain and urban runoff, losses from the supply and sewer network, and seawater, as demonstrated elsewhere (Vázquez-Suñé et al. 2010).

### Sample processing

Upon arrival of the samples at the laboratory, samples were filtered under vacuum through a 0.7-µm glass microfiber filter GF/F (Whatman). Ten milliliters of filtered samples were spiked with the internal standard mixture to achieve a final concentration of 50 μg L^−1^.

#### Protocol A

A 10-mL sample aliquot was concentrated using a VAE system (BÜCHI™ SyncorePlus, Switzerland) operating at 55 °C, 250 rpm orbital rotation, and a vacuum gradient to reach a final pressure of 20 mbar (Mechelke et al. 2019). The shape of the evaporation tube ensured a residual volume of 0.3 mL, which was then transferred to a 1.5 mL Eppendorf tube for centrifugation purposes (4000 rpm, 4 °C for 10 min). Then, the supernatant was transferred to an HPLC vial and evaporated under a gentle stream of N_2_ using a Pierce Reacti-Therm III (Thermo-Fisher Scientific, Germany). The sample was reconstituted in 500 µL ACN/H_2_O (95:5, v/v) for HILIC separation (Figure S1).

#### Protocol B

Following the same Protocol A enrichment process, once the VAE process ends, the residuals of 0.3 mL were transferred into an HPLC vial and 200 μL of H_2_O were added before RP chromatographic separation, obtaining a final sample volume of 500 µL (Figure S1).

### UPLC–Q Exactive Orbitrap-MS conditions and identification criteria

The two columns used in the study were an HSS T3 (100 × 2.1 mm, 1.8 µm particle size) and a BEH amide (100 × 2.1 mm, 1.7 µm), both from Waters (Milford, MA). The former contains a C18-modified package designed to retain nonpolar to moderately polar compounds, which generally fits with the physicochemical properties of the target analytes (New and Chan 2008). An ACQUITY UPLC system (Waters) was interfaced with a Q Exactive mass spectrometer (Thermo-Fisher Scientific, Germany) equipped with a heated electrospray ionization (HESI) probe. The system was controlled by Xcalibur 4.1 software and calibrated using a calibration solution. The LC conditions were optimized to achieve the best chromatographic separation. The flow rate for the HSS T3 column was set to 0.2 mL min^−1^, and mobile phases and further details can be found in (Gómez-Navarro et al. 2023). For the BEH amide column, the mobile phases consisted of (A) acetonitrile/water (98:2, v/v) with 5 mM ammonium formate and (B) 5 mM ammonium formate. The injection volume was 10 μL, and the column temperature was 40 °C. The optimized elution conditions for both columns are described in Table S3. Methanol was injected every six samples to check for any possible carryover or cross-contamination. In addition, to ensure the integrity of the analysis, quality control samples (QC) were prepared by spiking extracts of GW samples with standards and IS mixture (50 µg L^−1^) and were injected every six samples. For quantitative purposes, a 12-point calibration curve (0.05 to 300 µg L^−1^) was constructed containing all surrogates at 50 µg L^−1^.

The tuning methods and parameters used for the MS acquisitions were optimized. Moreover, a targeted-DIA experiment was performed to allow the acquisition of MS^2^ spectra only for ions defined in an inclusion list (Table S4) where the isolation window was set to 1.5 m/z, obtaining individual m/z and RT windows of high-quality MS^2^ spectra (Gómez-Navarro et al. 2023). Calibration was performed periodically in both electrospray ionization modes using two different high-purity mixtures from Thermo Fisher Scientific (Germany). The MS optimization was performed by injecting a standard solution containing all compounds at a concentration of 10 μg mL^−1^ in 100% H_2_O for the HSS T3 column and H_2_O/ACN (5:95, v/v) for the BEH amide.

Thermo Xcalibur software version 4.1 was used for instrument control and qualitative analysis. For quantitation of analytes in samples, data were processed using Thermo TraceFinder 5.1 software. For validation and quantification purposes, the Guidance SANTE 12682/2019 was followed (Pihlström et al. 2018).

### *Method validation*

For both protocols, A and B, three levels (1, 50, and 200 μg L^−1^) were used to assess the accuracy, precision, matrix effects, and limits of detection and quantification of all target analytes (Table S5 and S7). A total of 89 compounds were quantified in this study. The LOD ranged from 0.02 to 0.45 ng L^−1^, while LOQ ranged from 0.06 to 1.34 ng L^−1^ across the selected analytes. The calibration curve as well as all validation samples was prepared in matrix-matched groundwater. Regarding validation purposes, samples were prepared in triplicate and the matrix effect was corrected with the use of internal standards.

## Results and discussion

### Column performance

Given the wide range of target compound polarities, two stationary phase chemistries, namely, an RP C18 (HSS T3) and a HILIC (BEH amide) were tested in order to assess the suitability concerning retention factor and peak shape. The conditions offering an adequate compromise of separation efficiency and chromatographic runtime were a flow rate was 0.2 mL min^−1^ with a total analysis time of 19 and 15 min, respectively (Table S3). The capacity factor (RFK) was calculated according to the following equation:

1 $$RFK = ({t}_{r}- {t}_{0}) /{t}_{0}$$

where *t*_*r*_ is the retention time of the analyte and *t*_0_ is the elution time of an unretained compound (Table S4). The analyte providing *t*_0_ for the HSS T3 column was eflornithine (log *D* − 3.0) which was the first peak detectable without being retained in the column using the optimized gradient (*t*_*r*_ 0.88 min).

Comparing the two columns, there is a general trend between retention and polarity; in the HSS T3 column, low polar compounds are more retained, and in the BEH amide column is the opposite (Figure S2). For instance, using the HSS T3 column, some of the polar compounds studied, such as melamine, zanamivir, or 5-fluorouracil, showed poor retention (RFK < 1.5), combined with the peak shape quality of some of them such as metformin (Figure S3). The BEH amide column was also tested for the separation of 96 compounds to assess selectivity differences between the two columns. While eflornithine was the compound with no retention on the HSS T3 column, pentobarbital (log *D* 1.90) was the first detectable peak within the optimized HILIC gradient (*t*_*r*_ 0.91 min) and was selected as reference compound for RFK calculations. As an example, zanamivir was poorly retained in RP conditions (RFK 0.14) as was expected due to its high polarity (log *D* − 6.0). Using the BEH amide column helped retain and thus analyze this highly polar compound obtaining an RFK of 13.4. Other examples not related to polarity such as methenamine (log D 0.99) were also observed. This molecule was poorly retained in the HSS T3 column (RFK 0.11), whereas using the BEH amide column its retention increased to RFK 11.29.

**Fig. 2:**
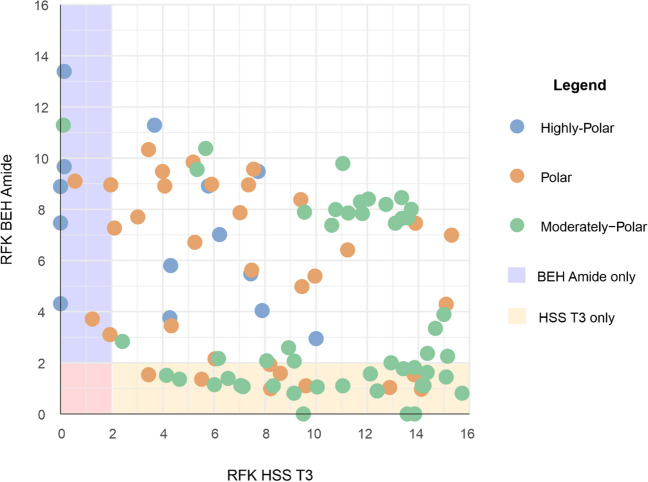
Relation between the target compounds retention on both columns based on their polarity classification. The red area of the graph shows the non-retained zone for both columns. The orange area shows where the compounds are better retained on the HSST3 column, while the blue zone shows the compounds only retained using BEH amide. The white area shows compounds well retained in both columns

Looking in-depth, a group of moderately polar compounds seems to have similar behavior, in both columns (Fig. 2). There are substances of this group that share similar properties, for example, propranolol, carazolol, and tramadol (log *D* of 1.15, 1.14, and 0.52, respectively) molecules sharing some functional groups as all three have 1 alcohol, 1 amine, and between 1 and 2 ethers, see Table S1 for structures. Following this pattern, metoprolol (log *D* − 0.3) was considered a polar compound, but it follows a similar behavior to the three molecules beforementioned and it could be mainly due to metoprolol having the same functional groups. Finally, atenolol could fit also with that pattern; however, atenolol contains an amide group at the end of the chain that makes it more polar (log *D* 1.9) and, as a result, its retention time in the reversed-phase column decreases considerably with respect the other four compounds. Eight more compounds inside this group could not be easily classified; there are citalopram, cocaine, hyoscine, neotame, oseltamivir, paroxetine, sitagliptin, and venlafaxine. From Fig. 2, it can also be observed that, above all, three highly polar compounds are well retained in both columns but more retained when using the HSS T3 column. These compounds are iopromide, sulisobenzone (BP4), and tetracycline (log *D* − 2.12, − 3.47, and − 3.20, respectively), all three large molecules (MW > 300 Da) with at least one aromatic group on their structures.

Additionally, the peak shape was used as a criterion for assessing the goodness of the separation (Kruve et al. 2015). Some compounds displayed peak shapes differing from Gaussian peaks in both columns (Figure S3). Thus, in order to evaluate the quality of each chromatographic peak taking into consideration the retention as well as the peak shape of each compound, a Quality Score (QS) was assigned for each analyte (Table 1). Here, a modification from (Iturrospe et al. 2021) was made, disregarding peak intensity in the overall assessment (Fig. 2).

**Table 1:** Quality Score system for each compound in both chromatographic columns

Peak shape score		Retention factor score	
0	No peak	0	No peak
1	Split peak or multiple peaks	1	RFK ≤ 1
4	Single peak with FWHM ≥ 2 or tailing factor ≥ 2 or ≤ 8	2	1 < RFK ≤ 2
7	Single peak with FWHM < 2 or tailing factor < 2 or > 8	3	RFK > 2

*FWHM*, full width at half maximum; *RFK*, retention factor.

**Fig. 3:**
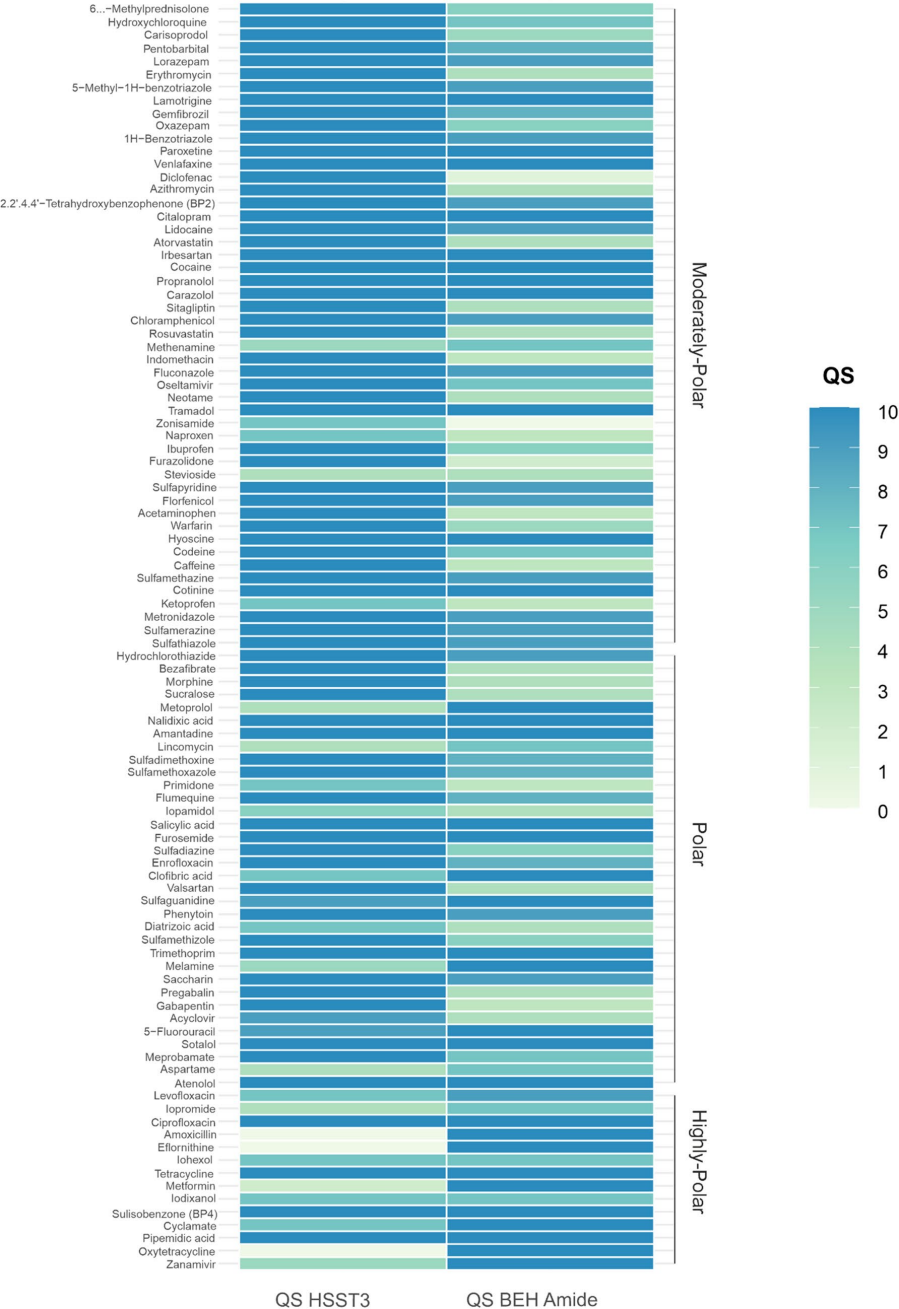
Quality Scores (QS) heatmap for each compound in both columns, sorted by polarity

Figure 3 shows the QS of each compound on both columns. In general terms, polar and moderately polar compounds demonstrate a great performance using the HSS T3 column. However, there are some exceptions such as aspartame (log *D* − 1.8), 5-fluorouracil (log *D* − 1.6), melamine (log *D* − 1.2), sulfaguanidine (log *D* − 1.0), clofibric acid (log *D* − 0.9), lincomycin (log *D* − 0.4), and metoprolol (log *D* − 0.3) that have higher QS in the HILIC column. That is mainly related to the peak shape of these substances. Among these substances, melamine was the one demonstrating better performance in both peak shape and retention under HILIC conditions (Figure S3). Except for 5-fluorouracil and sulfaguanidine, whose main difference between columns is based on retention and not on peak shape score, the other four compounds showed greater peak shape improvement using HILIC than using the HSS T3 column. Several mechanisms could affect the peak shape performance, but they are not completely understood yet. On the other hand, for the high-polar compounds, the BEH amide column results in a better performance compared to the HSS T3 column, which demonstrates its ability to retain and provide a Gaussian pattern for the polar compounds.

Given the distinct differences in QS of the analytes for the two column types, the 96 compounds were divided into two groups to ensure high chromatographic performance: 67 were analyzed on the RP column and 29 on the HILIC column (Table 2).

**Table 2:** Target compounds sorted by their polarity class and the chromatographic column selected for analysis

LC column	Polarity	Compounds
HSS T3	Polar	Acyclovir, bezafibrate, diatrizoic acid, enrofloxacin, flumequine, gabapentin, hydrochlorothiazide, meprobamate, morphine, phenytoin, pregabalin, primidone, saccharin, sucralose, sulfadiazine, sulfadimethoxine, sulfamethizole, sulfamethoxazole, valsartan
	Moderately polar	Acetaminophen, atorvastatin, azithromycin, BP2, caffeine, carazolol, chloramphenicol, citalopram, cocaine, codeine, cotinine, florfenicol, fluconazole, furazolidone, hyoscine, ibuprofen, indomethacin, irbesartan, ketoprofen, lidocaine, metronidazole, naproxen, neotame, oseltamivir, propranolol, rosuvastatin, sitagliptin, sulfamerazine, sulfamethazine, sulfapyridine, sulfathiazole, tramadol, warfarin, zonisamide, 1H-benzotriazole, 5-methyl-1H-lamotrigine, 6α-methylprednisolone, carisoprodol, diclofenac, erythromycin, gemfibrozil, hydroxychloroquine, lamotrigine, lorazepam, oxazepam, paroxetine, pentobarbital, venlafaxine
BEH amide	Highly polar	Amoxicillin, BP4, ciprofloxacin, cyclamate, eflornithine, iodixanol, iohexol, iopromide, levofloxacin, metformin, oxytetracycline, pipemidic acid, tetracycline, zanamivir
	Polar	5-Fluorouracil, amantadine, aspartame, atenolol, clofibric acid, furosemide, lincomycin, melamine, metoprolol, nalidixic acid, salicylic acid, sotalol, sulfaguanidine, trimethoprim
	Moderately polar	Methenamine

### Extraction of polar analytes

**Fig. 4:**
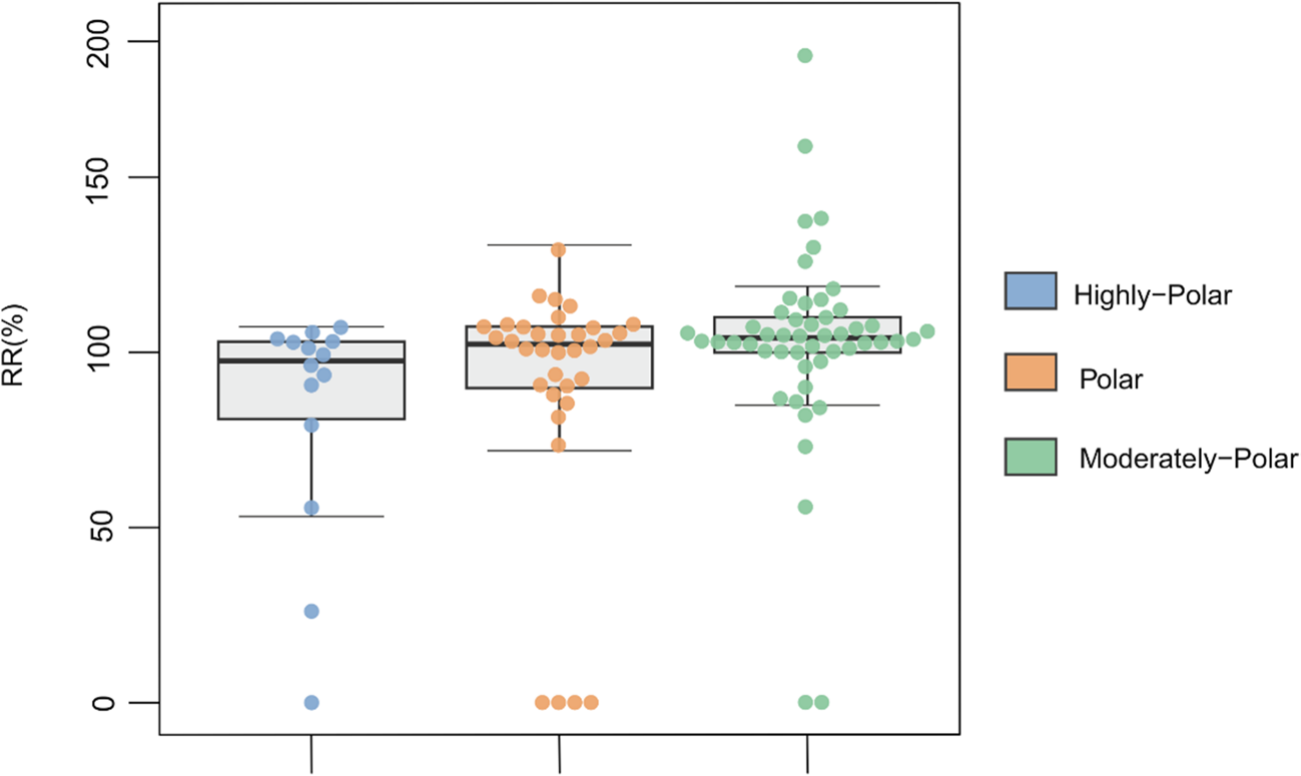
Relative VAE recoveries of the 96 analytes, expressed in percentage (RR %), as grouped by polarity class

As Fig. 4 shows, VAE enrichment provided a satisfactory performance for all three substance classes although there were substances in each class that were lost during the sample preparation. Analytes not recovered at all were codeine (moderately polar), naproxen (moderately polar), aspartame (polar), meprobamate (polar), sulfaguanidine (polar), morphine (polar), and amoxicillin (highly-polar). In the group of moderately polar compounds, tetracycline showed a suboptimal recovery of only 26%.

### Occurrence

**Fig. 5:**
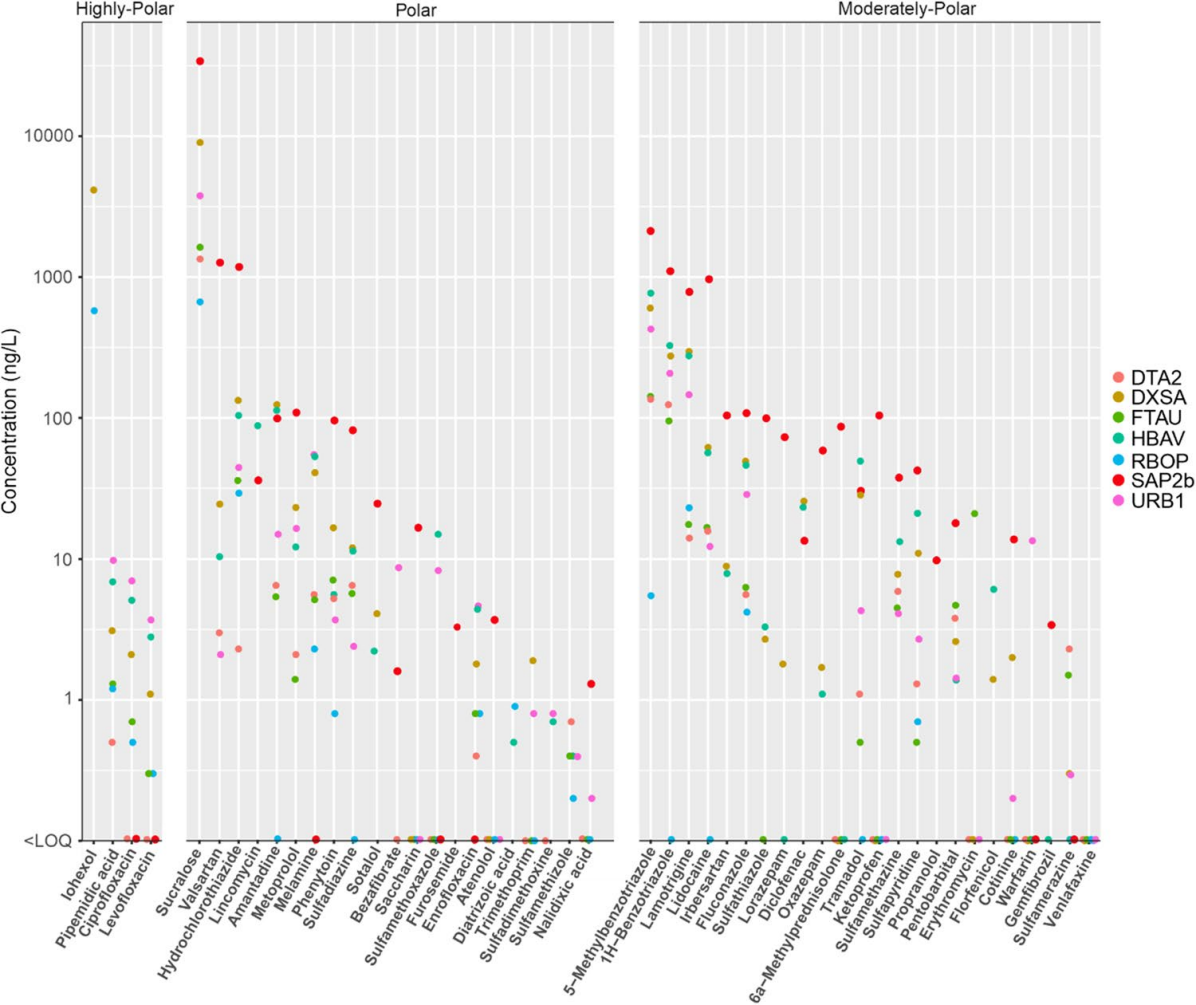
Concentrations of the analytes in the GW well samples collected in the vicinity of the Besos River. Compounds are grouped according to their polarity class

The analytical method was applied to GW well samples collected in the Besos River (Fig. 1) in order to assess the occurrence of the target analytes. The concentrations spanned a broad range of five orders of magnitude: from sub-ng L^−1^ to double-digit µg L^−1^ (Fig. 5). Then, 53 out of 89 were found above the LOD in at least one of the samples, while a total of 22 compounds were found in all seven GW samples above the LOD (Table S8). The sample SAP2b stood out insofar as the compound concentrations were generally higher than in the other samples because this well is directly impacted by the Besos River (Labad et al. 2023).

It is worth noting that the three fluoroquinolone antibiotics (ciprofloxacin, levofloxacin, and enrofloxacin) were detected in all GW well samples. They are used worldwide for the treatment of several kinds of bacterial infections in humans as well as in veterinary practice. A second group of antibiotics frequently detected were sulfonamides (sulfamethoxazole, sulfapyridine, sulfamerazine, and sulfadiazine). The concentrations of the drugs furosemide, gemfibrozil, hydrochlorothiazide, and valsartan exceed a concentration of 1 μg/L in sample SAP2b. Interestingly, furosemide and gemfibrozil were not measurable in any other sample. Previous studies of pharmaceuticals in groundwater in a pan-survey of wells from all over Europe provided max concentrations of some of them within the same range as detected in this study for sulfamethoxazole and diclofenac (Loos et al. 2010).

Other studies have demonstrated the presence of 5-methyl-1H-benzotriazole, sulfamethoxazole, ibuprofen, 1H-benzotriazole, and highly polar, e.g., melamine in groundwater and surface water in several across Europe and the US (Kruc et al. 2019; Loos et al. 2010; Neuwald et al. 2022; Schaider et al. 2014; Selak et al. 2022). There is a study regarding Barcelona wells made in 2013 (López-Serna et al. 2013), where 95 pharmaceuticals were analyzed and 33 are the same as our study. Similar zones (Besós deltaic area) were also studied. When compared, several agreements in both frequency of detection (FoD) and concentrations are observed. For example, the antibiotics sulfadiazine, sulfamethazine, and sulfamethoxazole showed very similar FoD as well as concentration ranges. Similar behaviors were observed with metoprolol, propranolol, and sotalol which are pharmaceuticals used to treat high blood pressure. Finally, hydrochlorothiazide, a diuretic, in both studies has been reported with a 100% FoD with similar minimum concentrations and close maximum concentrations (665 and 1179 ng L^−1^ in the López-Serna and the present study, respectively). On the other hand, there are some differences between studies, the main one is salicylic acid whose detection is opposite; not detected in the present study and fully detected in the comparison study. Diclofenac, ibuprofen, furosemide, and gemfibrozil are also compounds more detected and more concentrated in the 2013 study than in the present one. However, atenolol was detected in 85.7% of the samples here, while in the other study, it was detected just in one sample. Causes of agreement and disagreement in those molecules could be related to several factors, the main ones, being the year difference between studies, the season of the year, and, the obvious one, the wells are not the same in both studies.

Digging into the results, the substance with by far the highest measured concentration (34,100 ng L^−1^) was the artificial sweetener sucralose (log *D* − 0.2) whose average concentration across all wells was 7220 ng L^−1^. It has to be mentioned that in the sample point HBAV, sucralose was not detected. The presence of this chlorinated saccharose derivative in GW is a reflection of its extensive use in foodstuff, high water solubility and polarity, resistance to biodegradation over a wide range of pH, and very low affinity to soil particles. Sucralose was previously detected in two studies in non-urban groundwater from Barbados and Rastatt although low concentrations were found (Edwards et al. 2019; Wolf et al. 2012). Sweeteners, in general, are not included in legislation or watch lists due to their low toxicity.

Apart from these pharmaceuticals and artificial sweeteners, a series of pollutants considered of high environmental concern were found that are commonly classified as PMT (persistent—mobile—toxic) contaminants (1H-benzotriazole, 5-methyl-1H-benzotriazole, melamine, phenytoin, sulfadiazine, sulfapyridine, and venlafaxine) and as vPvM (very persistent—very mobile) contaminants (amantadine) (Hale et al. 2020). Among the PMT, the maximum concentrations of 5-methylbenzotriazole and 1H-benzotriazole were reached in sample Sap2b (2122 and 1100 ng L^−1^, respectively).

## Conclusions

In this study, the presence of pollutants with a wide range of polarities in the urban GW of Barcelona city was evaluated. Through the calculation of the QS of each compound on both columns, we can conclude that moderately polar compounds were satisfactorily separated on a C18 column (HSS T3). However, the chromatography of highly polar substances was more challenging due to their poor retention so aspartame, clofibric acid, 5-fluorouracil, melamine, lincomycin, metoprolol, and sulfaguanidine presented higher QS in the HILIC column. That is mainly related to the peak shape of these substances. Finally, the VAE enrichment combined with the chromatographic separation on both columns provided the best performance.

The analytical protocol was applied to real samples from GW wells showing that GW is not only polluted with compounds previously detected but also with highly polar and polar compounds including artificial sweeteners, industrial chemicals, and pharmaceuticals. The concentrations of most of the organic contaminants in GW were less than 100 ng L^−1^. A notable exception was the artificial sweetener sucralose with a peak concentration of 34,100 ng L^−1^ as well as the highly polar compound, iohexol, with concentrations higher than 1000 ng L^−1^. The study highlighted a clear need for updating existing monitoring schemes to include very polar substances, which will allow to monitor GW quality in a more comprehensive matter and thus assess potential drinking water sources.

## Supplementary Information

Below is the link to the electronic supplementary material.

Supplementary file1 (DOCX 1289 KB)

## Declarations

### Disclaimer

The EU is not liable for any use that may be made of the information contained therein.
