# Supplementary material for: LC–MS analysis of polar and highly polar organic pollutants in Barcelona urban groundwater using orthogonal LC separation modes
Source: Environ Sci Pollut Res Int. 2024 Apr 26;33(22):11018–28. doi: 10.1007/s11356-024-33471-y (PMC13415668; doi:10.1007/s11356-024-33471-y)
Supplement: Supplementary file 1 — Supplementary file1 (DOCX 1289 KB) [file 11356_2024_33471_MOESM1_ESM.docx]

**Supplementary Data**

**LC-MS analysis of polar and highly polar organic pollutants in Barcelona urban groundwater using orthogonal LC separation modes**

Francesc Labad^1^, Sandra Pérez^1*^

^1^ONHEALTH, Department of Environmental Chemistry, Institute of Environmental Assessment and Water Research (IDAEA), CSIC, Jordi Girona 18-26, 08034 Barcelona (Spain)

****Corresponding author:***

Sandra Pérez

IDAEA-CSIC

ONHEALTH Research Group

Jordi Girona 18-26

Barcelona 08034, Spain

E-mail: spsqam@idaea.csic.es

Phone: +34-93 400 6100

Fax: +34-93 204 5904

**Table of contents**

[Table S1. List of the 96 target compounds sorted by log*D*^1^ 23](#_Toc160795102)

[Table S2. In situ parameters of the seven GW samples. 37](#_Toc160795103)

[Figure S1. Flow-chart for enrichment by VAE followed by HILIC (left) and C18 (right) chromatographic separation before HPLC-MS/MS analysis. 38](#_Toc160795104)

[Figure S2. Retention differences between columns and compounds polarities. 39](#_Toc160795105)

[Figure S3. A: Chromatograms of diclofenac, atorvastatin and gabapentin; B: Chromatograms of melamine, metformin and methenamine. A shows great performance using the HSS T3 column (Upper) while B shows great BEH Amide column (Lower) performance for the selected compounds shown. 40](#_Toc160795106)

[Table S3. Parameters of the optimised gradients. 41](#_Toc160795107)

[Table S4. MS parameters, chromatographic retention times (RT) and retention factors (RFK) of target compounds sorted by alphabetical order. The t_0_ for the HSS T3 column was 0.90 min. 42](#_Toc160795108)

[Table S5. Validation parameters of the target compounds enriched by the VAE approach at 1, 50 and 200 µg/L (n = 3), sorted by alphabetical order. 48](#_Toc160795109)

[Table S6. Sample location in coordinates. 53](#_Toc160795110)

[Table S7. Limits of detection (LOD) and quantification (LOQ) of the target analytes, sorted by alphabetical order. 54](#_Toc160795135)

[Table S8. Results of the seven wells samples studied sorted by Log D (pH 7.4). The concentration values shown are in ng L^-1^. 57](#_Toc160795136)

[Table S9. Quality Score for each compound in both columns, sorted by log D. 60](#_Toc160795137)

# Table S1. List of the 96 target compounds sorted by log*D*

| Compound | Main use | CAS | Molecular formula | Molecular weight | Water Solubility^1^  (mg mL^-1^) | Boiling point^1^  (°C) | Log D^1^ at pH 7.4 | Structure |
| --- | --- | --- | --- | --- | --- | --- | --- | --- |
| Zanamivir | Antiviral | 1391180-80-8 | C_12_H_20_N_4_O_7_ | 332.31 | 18 | - | -6.0 |  |
| Oxytetracycline | Antibiotic | 79-57-2 | C_22_H_24_N_2_O_9_ | 460.40 | 31 | 840 | -4.3 |  |
| Pipemidic acid | Antibiotic | 51940-44-4 | C_14_H_17_N_5_O_3_ | 303.32 | 0.32 | 535 | -3.6 |  |
| Cyclamate | Sweetener | 100-88-9 (cyclamic acid) | C_6_H_13_NO_3_S | 179.24 | 133 | - | -3.5 |  |
| Sulisobenzone (BP4) | UV-Filter | 4065-45-6 | C_14_H_12_O_6_S | 308.31 | 250 | 498 | -3.5 |  |
| Metformin | Diabetes type 2 treatment agent | 657-24-9 | C_4_H_11_N_5_ | 129.16 | 300 | 173 | -3.4 |  |
| Iodixanol | Iodinated contrast media | 92339-11-2 | C_35_H_44_I_6_N_6_O_15_ | 1550.20 | 0.19 | 1251 | -3.4 |  |
| Tetracycline | Antibiotic | 60-54-8 | C_22_H_24_N_2_O_8_ | 444.40 | 0.23 | 738 | -3.2 |  |
| Iohexol | Iodinated contrast media | 66108-95-0 | C_19_H_26_I_3_N_3_O_9_ | 821.14 | 350 | 892 | -3.2 |  |
| Eflornithine | Facial hirsutism treatment drug | 70052-12-9 | C_6_H_12_F_2_N_2_O_2_ | 182.17 | 10 | 347 | -3.0 |  |
| Amoxicillin | Antibiotic | 26787-78-0 | C_16_H_19_N_3_O_5_S | 365.40 | 0.96 | 743 | -2.7 |  |
| Ciprofloxacin | Antibiotic | 85721-33-1 | C_17_H_18_FN_3_O_3_ | 331.35 | 1.4 | 582 | -2.2 |  |
| Levofloxacin | Antibiotic | 100986-85-4 | C_18_H_20_FN_3_O_4_ | 361.40 | 0.05 | 572 | -2.1 |  |
| Iopromide | Iodinated contrast media | 73334-07-3 | C_18_H_24_I_3_N_3_O_8_ | 791.11 | 0.37 | 841 | -2.1 |  |
| Atenolol | β-blocker  agent | 29122-68-7 | C_14_H_22_N_2_O_3_ | 266.34 | 13 | 508 | -1.9 |  |
| Aspartame | Sweetener | 22839-47-0 | C_14_H_18_N_2_O_5_ | 294.30 | 10 | 536 | -1.8 |  |
| Meprobamate | Anxiolytic | 57-53-4 | C_9_H_18_N_2_O_4_ | 218.25 | 2.5 | 334 | -1.8 |  |
| Sotalol | β-blocker  agent | 3930-20-9 | C_12_H_20_N_2_O_3_S | 272.36 | 0.78 | 443 | -1.6 |  |
| 5-Fluorouracil | Antineoplastic agent | 51-21-8 | C_4_H_3_FN_2_O_2_ | 130.08 | 5.9 | 367 | -1.6 |  |
| Acyclovir | Antiviral | 59277-89-3 | C_8_H_11_N_5_O_3_ | 225.20 | 9.1 | 500 | -1.5 |  |
| Gabapentin | Anticonvulsant | 60142-96-3 | C9H17NO2 | 171.24 | 4.5 | 314 | -1.4 |  |
| Pregabalin | Anticonvulsant | 148553-50-8 | C_8_H_17_NO_2_ | 159.23 | 11 | 274 | -1.3 |  |
| Saccharin | Sweetener | 81-07-2 | C_7_H_5_NO_3_S | 183.18 | 4.0 | 389 | -1.3 |  |
| Melamine | Industrial compound | 108-78-1 | C_3_H_6_N_6_ | 126.12 | 3.2 | 558 | -1.2 |  |
| Trimethoprim | Antibiotic | 738-70-5 | C_14_H_18_N_4_O_3_ | 290.32 | 0.62 | 405 | -1.2 |  |
| Sulfamethizole | Antibiotic | 144-82-1 | C_9_H_10_N_4_O_2_S_2_ | 270.33 | 0.61 | 505 | -1.1 |  |
| Diatrizoic acid | Iodinated contrast media | 117-96-4 | C_11_H_9_I_3_N_2_O_4_ | 613.91 | 0.11 | 614 | -1.0 |  |
| Sulfaguanidine | Antibiotic | 57-67-0 | C_7_H_10_N_4_O_2_S | 214.25 | 0.81 | 426 | -1.0 |  |
| Phenytoin | Anticonvulsant | 57-41-0 | C_15_H_12_N_2_O_2_ | 252.27 | 0.07 | 464 | -1.0 |  |
| Clofibric acid | Lipid-lowering agent | 882-09-7 | C_10_H_11_ClO_3_ | 214.64 | - | 324 | -0.9 |  |
| Enrofloxacin | Antibiotic | 93106-60-6 | C_19_H_22_FN_3_O_3_ | 359.40 | 0.05 | 561 | -0.9 |  |
| Valsartan | Antihypertensive | 137862-53-4 | C_24_H_29_N_5_O_3_ | 435.52 | 0.02 | 685 | -0.9 |  |
| Furosemide | Antihypertensive | 54-31-9 | C_12_H_11_ClN_2_O_5_S | 330.74 | 0.07 | 582 | -0.8 |  |
| Salicylic acid | Antibacterial | 69-72-7 | C_7_H_6_O_3_ | 138.12 | 2.24 | 336 | -0.8 |  |
| Sulfadiazine | Antibiotic | 68-35-9 | C_10_H_10_N_4_O_2_S | 250.28 | 0.6 | 513 | -0.8 |  |
| Flumequine | Antibiotic | 42835-25-6 | C_14_H_12_FNO_3_ | 261.25 | <1.0 | 440 | -0.7 |  |
| Primidone | Anticonvulsant | 125-33-7 | C_12_H_14_N_2_O_2_ | 218.25 | 1.0 | 443 | -0.7 |  |
| Sulfamethoxazole | Antibiotic | 723-46-6 | C_10_H_11_N_3_O_3_S | 253.28 | 0.46 | 482 | -0.6 |  |
| Sulfadimethoxine | Antibiotic | 122-11-2 | C_12_H_14_N_4_O_4_S | 310.33 | 0.34 | 549 | -0.5 |  |
| Amantadine | Antiviral | 768-94-5 | C_10_H_17_N | 151.14 | 6.3 | 226 | -0.4 |  |
| Lincomycin | Antibiotic | 154-21-2 | C_18_H_34_N_2_O_6_S | 406.54 | - | 647 | -0.4 |  |
| Metoprolol | β-blocker  agent | 37350-58-6 | C_15_H_25_NO_3_ | 267.36 | >1000 | 399 | -0.3 |  |
| Nalidixic acid | Antibiotics | 389-08-2 | C_12_H_12_N_2_O_3_ | 232.24 | 2.3 | 413 | -0.3 |  |
| Sucralose | Sweetener | 56038-13-2 | C_12_H_19_Cl_3_O_8_ | 397.63 | >1000 | 669 | -0.2 |  |
| Morphine | Analgesic | 57-27-2 | C_17_H_19_NO_3_ | 285.34 | 10 | 476 | -0.1 |  |
| Bezafibrate | Lipid-lowering agent | 41859-67-0 | C_19_H_20_ClNO_4_ | 361.82 | 2*10^-3^ | 572 | -0.1 |  |
| Hydrochlorothiazide | Antihypertensive | 58-93-5 | C_7_H_8_ClN_3_O_4_S_2_ | 297.74 | 0.72 | 577 | 0.0 |  |
| Sulfathiazole | Antibiotic | 72-14-0 | C_9_H_9_N_3_O_2_S_2_ | 255.32 | 0.37 | 480 | 0.0 |  |
| Sulfamerazine | Antibiotic | 127-79-7 | C_11_H_12_N_4_O_2_S | 264.30 | 0.20 | 519 | 0.0 |  |
| Metronidazole | Antibacterial | 443-48-1 | C_6_H_9_N_3_O_3_ | 171.15 | 5.9 | 405 | 0.1 |  |
| Ketoprofen | NSAID | 22071-15-4 | C_16_H_14_O_3_ | 254.28 | 0.02 | 431 | 0.1 |  |
| Cotinine | Stimulant | 486-56-6 | C_10_H_12_N_2_O | 176.22 | - | 329 | 0.2 |  |
| Sulfamethazine | Antibiotics | 57-68-1 | C_12_H_14_N_4_O_2_S | 278.33 | 0.23 | 526 | 0.2 |  |
| Hyoscine | Anticholinergic | 149-64-4 | C_21_H_29_NO_4_ | 359.46 | 100 | 460 | 0.3 |  |
| Caffeine | Stimulant | 58-08-2 | C_8_H_10_N_4_O_2_ | 194.19 | 22 | 417 | 0.3 |  |
| Codeine | Analgesic | 76-57-3 | C_18_H_21_NO_3_ | 299.36 | 0.58 | 462 | 0.3 |  |
| Warfarin | Anticoagulant | 81-81-2 | C_19_H_16_O_4_ | 308.30 | 0.02 | 515 | 0.3 |  |
| Sulfapyridine | Antibiotic | 144-83-2 | C_11_H_11_N_3_O_2_S | 249.29 | 0.27 | 474 | 0.4 |  |
| Florfenicol | Antibiotic | 73231-34-2 | C_12_H_14_C_l2_FNO_4_S | 358.21 | - | - | 0.4 |  |
| Acetaminophen | Analgesic | 103-90-2 | C_8_H_9_NO_2_ | 151.16 | 14 | 388 | 0.4 |  |
| Furazolidone | Antibiotics | 67-45-8 | C_8_H_7_N_3_O_5_ | 225.16 | 0.36 | 353 | 0.4 |  |
| Naproxen | NSAID | 22204-53-1 | C_14_H_14_O_3_ | 230.26 | 0.02 | 404 | 0.5 |  |
| Zonisamide | Anticonvulsant | 68291-97-4 | C_8_H_8_N_2_O_3_S | 212.23 | 2.1 | 457 | 0.5 |  |
| Ibuprofen | NSAID | 15687-27-1 | C_13_H_18_O_2_ | 206.28 | 0.02 | 320 | 0.5 |  |
| Tramadol | Analgesic | 3715-90-0 | C_13_H_18_ClN_3_ | 251.75 | 0.75 | 388 | 0.5 |  |
| Oseltamivir | Antiviral | 196618-13-0 | C_16_H_28_N_2_O_4_ | 312.40 | 0.69 | 473 | 0.6 |  |
| Neotame | Sweetener | 165450-17-9 | C_20_H_30_N_2_O_5_ | 378.46 | 0.01 | 572 | 0.6 |  |
| Fluconazole | Antifugals | 86386-73-4 | C_13_H_12_F_2_N_6_O | 306.27 | 1.4 | 580 | 0.7 |  |
| Indomethacin | NSAID | 53-86-1 | C_19_H_16_ClNO_4_ | 357.79 | 0.9*10^-3^ | 499 | 0.8 |  |
| Rosuvastatin | NSAID | 287714-41-4 | C_22_H_28_FN_3_O_6_S | 481.54 | 0.09 | 746 | 1.0 |  |
| Chloramphenicol | Antibiotic | 56-75-7 | C_11_H_12_Cl_2_N_2_O_5_ | 323.13 | 2.5 | 645 | 1.0 |  |
| Methenamine | Antibacterial | 100-97-0 | C_6_H_12_N_4_ | 140.19 | 449 | 253 | 1.0 |  |
| Sitagliptin | Diabetes type 2 treatment | 486460-32-6 | C_16_H_15_F_6_N_5_O | 407.31 | 0.03 | 530 | 1.1 |  |
| Carazolol | β-blocker  agent | 57775-29-8 | C_18_H_22_N_2_O_2_ | 298.38 | 0.01 | 469 | 1.1 |  |
| Propranolol | β-blocker  agent | 525-66-6 | C_16_H_21_NO_2_ | 259.34 | 0.06 | 435 | 1.2 |  |
| Cocaine | Stimulant | 50-36-2 | C_17_H_21_NO_4_ | 303.35 | 1.8 | 395 | 1.2 |  |
| Irbesartan | Antihypertensive | 138402-11-6 | C_25_H_28_N_6_O | 428.53 | 0.01 | 649 | 1.2 |  |
| Atorvastatin | Lipid-lowering agent | 134523-00-5 | C_33_H_35_FN_2_O_5_ | 558.60 | <1 | 722 | 1.3 |  |
| Citalopram | Antidepressant | 59729-33-8 | C_20_H_21_FN_2_O | 324.16 | 0.02 | 352 | 1.3 |  |
| Lidocaine | Anesthetic | 137-58-6 | C_14_H_22_N_2_O | 234.34 | 0.59 | 373 | 1.3 |  |
| Azithromycin | Antibiotic | 83905-01-5 | C_38_H_72_N_2_O_12_ | 748.98 | - | 822 | 1.4 |  |
| Venlafaxine | Antidepressant | 93413-69-5 | C_17_H_27_NO_2_ | 277.40 | 572 | 398 | 1.4 |  |
| Diclofenac | NSAID | 15307-86-5 | C_14_H_11_Cl_2_NO_2_ | 296.15 | 2.3*10^-3^ | 412 | 1.4 |  |
| 2.2'.4.4'-Tetrahydroxybenzophenone (BP2) | UV-Filter | 131-55-5 | C_13_H_10_O_5_ | 246.22 | 0.01 | 559 | 1.4 |  |
| 1H-Benzotriazole | Industrial compound | 95-14-7 | C_6_H_5_N_3_ | 119.12 | 20 | 359 | 1.5 |  |
| Oxazepam | Antidepressant | 604-75-1 | C_15_H_11_N_2_O_2_Cl | 286.71 | 0.02 | 517 | 1.5 |  |
| Paroxetine | Antidepressant | 61869-08-7 | C_19_H_20_FNO_3_ | 329.37 | 1.1*10^-3^ | 452 | 1.5 |  |
| Gemfibrozil | Lipid-lowering drug | 25812-30-0 | C_15_H_22_O_3_ | 250.33 | 0.03 | 395 | 1.6 |  |
| Erythromycin | Antibiotic | 114-07-8 | C_37_H_67_NO_13_ | 733.93 | 2.0 | 720 | 1.7 |  |
| 5-Methyl-1H-benzotriazole | Industrial compound | 136-85-6 | C_7_H_7_N_3_ | 133.15 | 3.1 | 211 | 1.7 |  |
| Lamotrigine | Antidepressant | 84057-84-1 | C_9_H_7_Cl_2_N_5_ | 256.09 | 0.17 | 503 | 1.7 |  |
| Carisoprodol | Muscle relaxant | 78-44-4 | C_12_H_24_N_2_O_4_ | 260.33 | 0.30 | 423 | 1.9 |  |
| Pentobarbital | Sedative | 76-74-4 | C_11_H_18_N_2_O_3_ | 226.28 | 0.68 | - | 1.9 |  |
| Lorazepam | Anxiolytic | 846-49-1 | C_15_H_10_Cl_2_N_2_O_2_ | 321.20 | 0.08 | 534 | 1.9 |  |
| Hydroxychloroquine | Antimalarial drug | 118-42-3 | C_18_H_26_ClN_3_O | 335.87 | 0.03 | 517 | 2.0 |  |
| 6∝-Methylprednisolone | Antiinflammatory | 83-43-2 | C_22_H_30_O_5_ | 374.47 | 0.12 | 572 | 2.0 |  |

^1^Log D, solubility and boiling point values were obtained from the ChemSpider database <http://www.chemspider.com/>.

#

# Table S2. In situ parameters of the seven GW samples.

| **Site** | **pH** | **EC^1^ (μS cm^-1^)** | **T (⁰C)** | **Hardness (mg L^-1^)** |
| --- | --- | --- | --- | --- |
| HBAV | 6.7 | 1163 | 25.0 | 305.2 |
| DXSA | 6.9 | 1270 | 19.7 | 291.2 |
| RBOP | 5.5 | 580 | 21.8 | 201.2 |
| URB1 | 6.7 | 1422 | 19.7 | 375.2 |
| SAP2b | 6.9 | 2541 | 21.3 | 537.6 |
| DTA2 | 6.8 | 1730 | 20.1 | 479.2 |
| FTAU | 6.6 | 1699 | 21.5 | 429.6 |

^1^Electric conductivity.


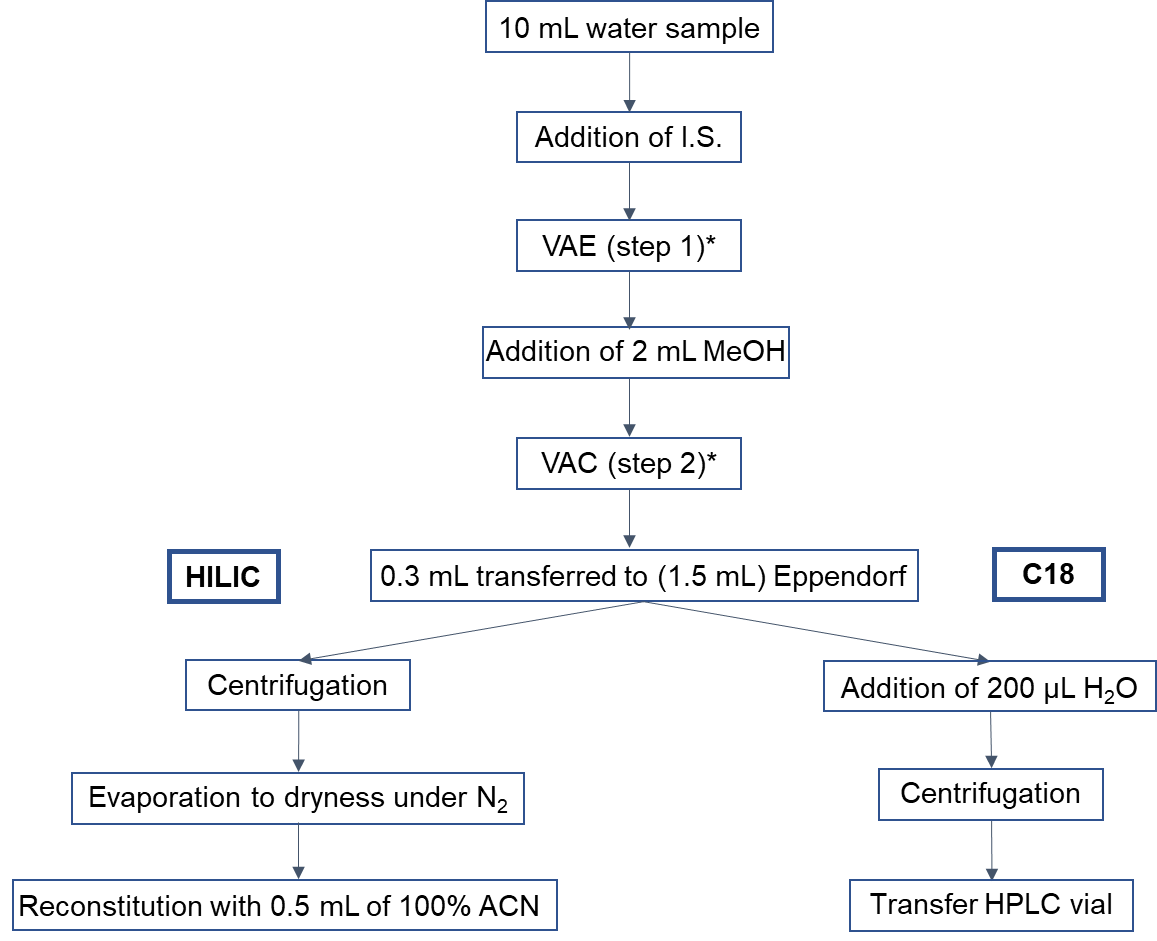


* For further information see Mechelke et al. (Mechelke et al., 2019).

# Figure S1. Flow-chart for enrichment by VAE followed by HILIC (left) and C18 (right) chromatographic separation before HPLC-MS/MS analysis.

#
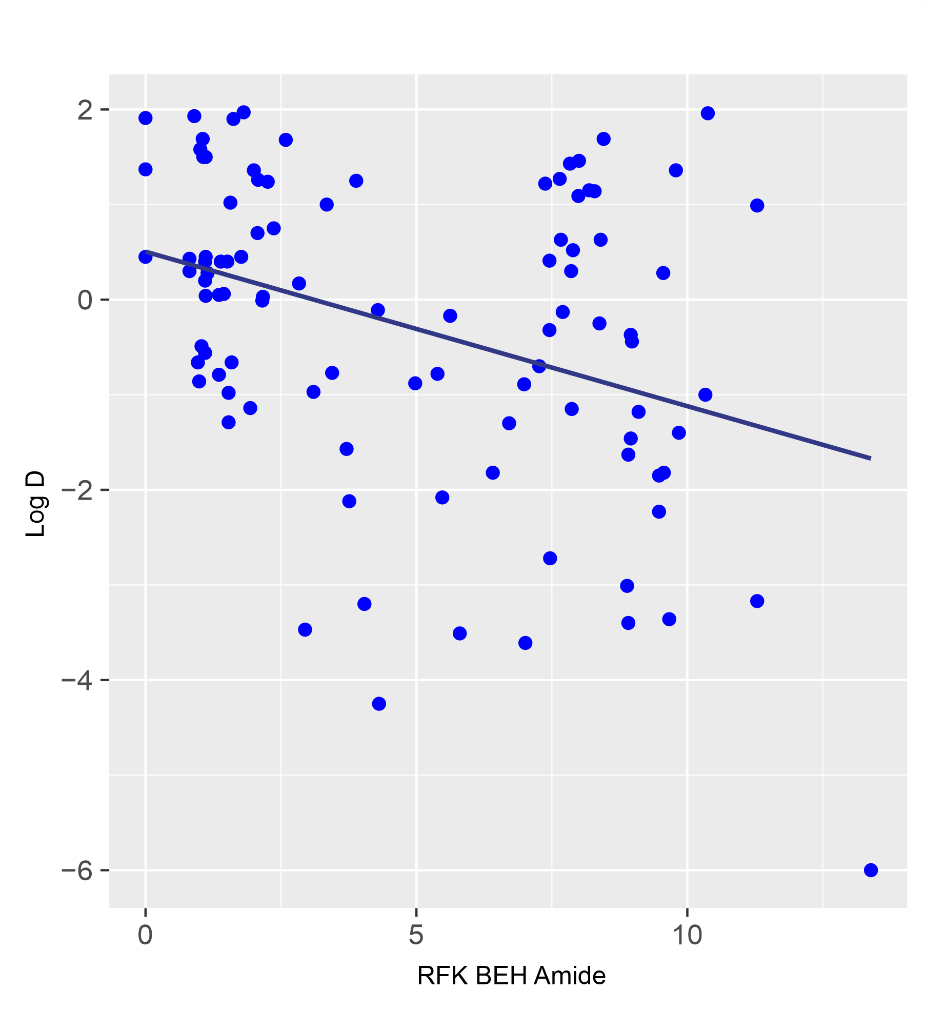

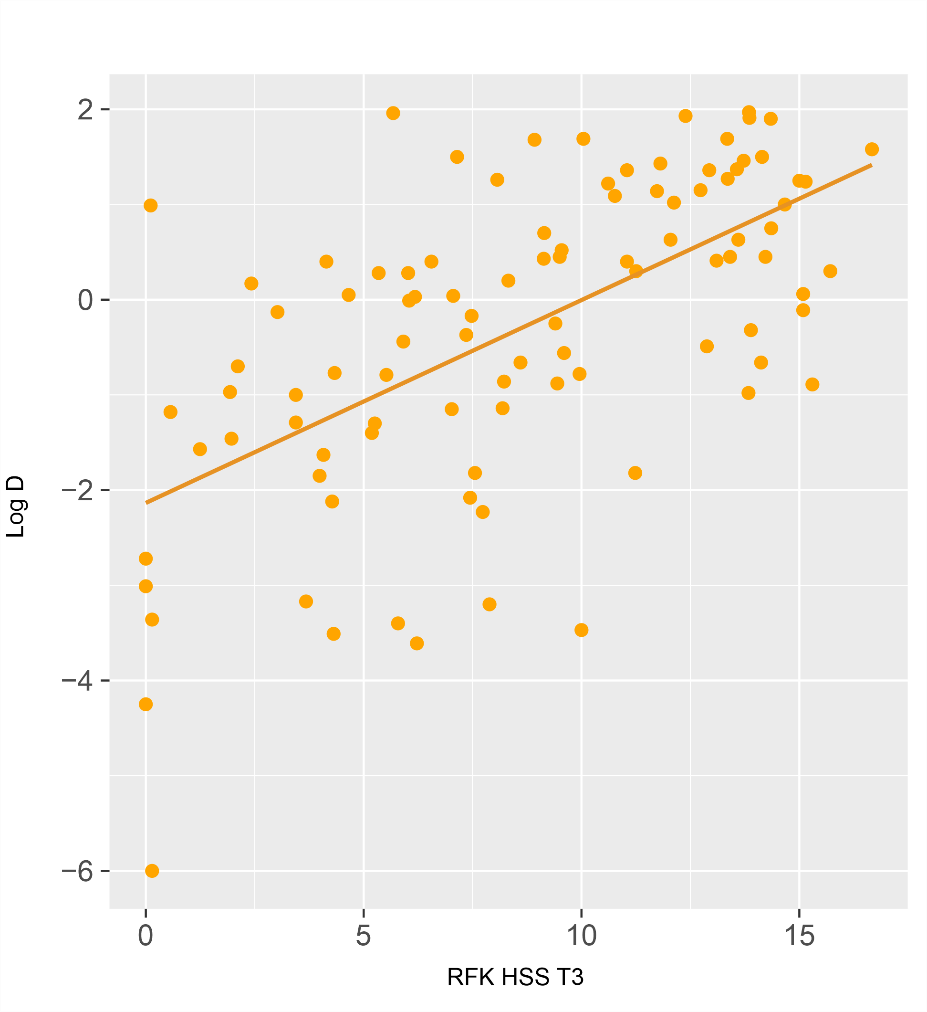
Figure S2. Retention differences between columns and compounds polarities.

**
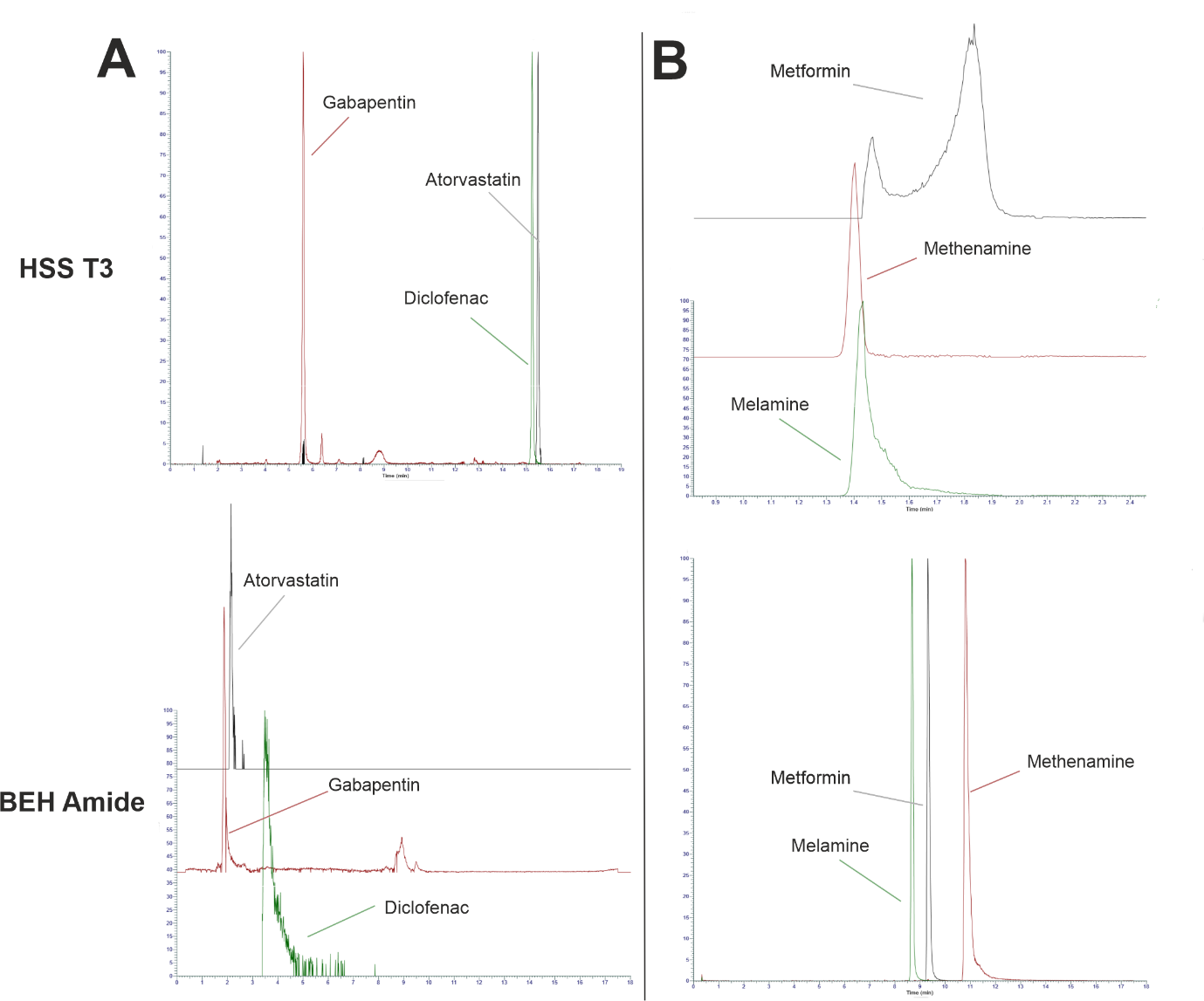
**

# Figure S3. A: Chromatograms of diclofenac, atorvastatin and gabapentin; B: Chromatograms of melamine, metformin and methenamine. A shows great performance using the HSS T3 column (Upper) while B shows great BEH Amide column (Lower) performance for the selected compounds shown.

# Table S3. Parameters of the optimised gradients.

| **Optimised Gradient HSS T3** | | | |
| --- | --- | --- | --- |
| Flow (mL/min) | Time (min) | Solvent A* (%) | Solvent B* (%) |
| 0.2 | 0 | 5 | 95 |
| 0.2 | 0.3 | 5 | 95 |
| 0.2 | 10 | 30 | 70 |
| 0.2 | 13.3 | 65 | 35 |
| 0.2 | 15.5 | 100 | 0 |
| 0.2 | 17.3 | 100 | 0 |
| 0.2 | 17.7 | 5 | 95 |
| 0.2 | 19 | 5 | 95 |

| **Optimised Gradient HILIC** | | | |
| --- | --- | --- | --- |
| Flow (mL/min) | Time (min) | Solvent A** (%) | Solvent B** (%) |
| 0.2 | 0 | 100 | 0 |
| 0.2 | 1 | 100 | 0 |
| 0.2 | 8 | 50 | 50 |
| 0.2 | 11 | 50 | 50 |
| 0.2 | 12 | 100 | 0 |
| 0.2 | 15 | 100 | 0 |

* A (ESI+): H_2_O 5mM ammonium formate 0.1% formic acid

A (ESI-): H_2_O 2mM ammonium fluoride

B (ESI+/-): 100% ACN

** A (ESI+/-): H_2_O 5mM ammonium formate

B (ESI+/-): H_2_O:ACN (2:98, v/v) 5mM ammonium formate

# Table S4. MS parameters, chromatographic retention times (RT) and retention factors (RFK) of target compounds sorted by alphabetical order. The t_0_ for the HSS T3 column was 0.90 min.

| **Analyte** | **ESI mode** | **Precursor (m/z)** | **Product Ion (m/z)** | **CE (eV)** | **RT (min)** | **RFK** | **Internal Standard** |
| --- | --- | --- | --- | --- | --- | --- | --- |
| 1H-Benzotriazole | + | 120.0557 | 65.0387 | 20 | 7.33 | 7.14 | Benzotriazole D4 |
| 2,2',4,4'-Tetrahydroxybenzophenone (BP2) | - | 245.0454 | 109.0294 | 19 | 12.54 | 12.93 | Irbesartan D6 |
| 5-Fluorouracil | - | 129.0106 | 85.0290 | 37 | 2.02 | 1.24 | Gabapentin D10 |
| 5-methyl-1H-benzotriazole | + | 134.0712 | 79.0542 | 25 | 9.94 | 10.04 | Cotinine D3 |
| 6αmethylprednisolone | + | 375.2162 | 357.2063 | 22 | 13.36 | 13.84 | Paroxetine D4 |
| Acetaminophen | + | 152.0706 | 110.0602 | 20 | 4.63 | 4.14 | Acetaminophen D4 |
| Acyclovir | - | 224.0789 | 150.0419 | 35 | 2.67 | 1.97 | Gabapentin D10 |
| Amantadine | + | 152.1431 | 135.1167 | 15 | 7.52 | 7.36 | Aspartame D5 |
| Amoxicillin | + | 366.1118 | 114.0008 | 12 | 3.45 | 2.83 | Cotinine D3 |
| Aspartame | + | 295.1292 | 180.1019 | 25 | 7.70 | 7.56 | Sotalol D6 |
| Atenolol | + | 267.1701 | 190.0861 | 25 | 4.49 | 3.99 | Propranolol D7 |
| Atorvastatin | + | 559.2603 | 440.2248 | 38 | 14.40 | 15.00 | Climbazole D4 |
| Azithromycin | + | 749.5146 | 591.4214 | 44 | 10.84 | 11.04 | Ciprofloxacin D8 |
| Bezafibrate | + | 362.1147 | 316.1096 | 14 | 14.48 | 15.09 | Bezafibrate D4 |
| Caffeine | + | 195.0877 | 138.0664 | 27 | 6.32 | 6.02 | Caffeine 13C3 |
| Carazolol | + | 299.1754 | 116.1068 | 40 | 11.46 | 11.73 | Propranolol D7 |
| Carisoprodol | + | 261.1805 | 62.0238 | 20 | 12.05 | 12.39 | Carisoprodol D7 |
| Chloramphenicol | - | 321.0045 | 152.0352 | 13 | 11.81 | 12.12 | Irbesartan D6 |
| Ciprofloxacin | + | 332.1403 | 288.1508 | 19 | 7.86 | 7.73 | Ciprofloxacin D8 |
| Citalopram | + | 325.1706 | 109.0446 | 26 | 12.92 | 13.36 | Midazolam 13C6 |
| Clofibric acid | - | 213.0323 | 126.9956 | 10 | 9.40 | 9.44 | Bezafibrate D4 |
| Cocaine | + | 304.1538 | 182.1174 | 30 | 10.45 | 10.61 | Cocaine D5 |
| Codeine | + | 300.1597 | 165.0697 | 42 | 5.71 | 5.34 | Codeine D3 |
| Cotinine | + | 177.1021 | 80.0494 | 28 | 3.08 | 2.42 | **Cotinine** D3 |
| Cyclamate | - | 178.0543 | 79.9577 | 34 | 4.78 | 4.31 | Acetaminophen D4 |
| Diatrizoic acid | + | 631.8031 | 360.9680 | 25 | 4.00 | 3.44 | Metronidazole D4 |
| Diclofenac | + | 296.0245 | 215.0496 | 30 | 13.11 | 13.57 | Diclofenac 13C6 |
| Eflornithine | + | 183.0940 | 120.1000 | 25 | 0.90 | 0.00 | Cotinine D3 |
| Enrofloxacin | + | 360.1723 | 316.1824 | 28 | 8.30 | 8.22 | Lamotrigine 13C3 |
| Erythromycin | + | 734.4666 | 158.1174 | 29 | 12.91 | 13.34 | Ciprofloxacin D8 |
| Florfenicol | - | 355.9927 | 119.0501 | 14 | 10.84 | 11.04 | Sulfamethoxazole 13C6 |
| Fluconazole | - | 305.0966 | 68.0252 | 12 | 9.13 | 9.14 | Fluconazole 13C3 |
| Flumequine | + | 262.0874 | 244.0766 | 30 | 13.61 | 14.12 | Carbamazepine D10 |
| Furazolidone | + | 226.0458 | 95.0366 | 18 | 9.12 | 9.13 | Fluconazole 13C3 |
| Furosemide | - | 329.0004 | 204.9823 | 30 | 9.86 | 9.96 | Valsartan D3 |
| Gabapentin | + | 172.1337 | 154.1226 | 17 | 5.57 | 5.19 | Gabapentin D10 |
| Gemfibrozil | - | 249.1496 | 121.0659 | 14 | 15.90 | 16.67 | Gemfibrozil D6 |
| Hydrochlorothiazide | - | 295.9568 | 268.9457 | 29 | 6.34 | 6.04 | Hydrochlorothiazide 13CD2 |
| Hydroxychloroquine | + | 336.1835 | 247.0996 | 30 | 6.01 | 5.68 | Chlorpromazine D3 |
| Hyoscine | + | 360.2164 | 138.0913 | 30 | 11.03 | 11.26 | Cocaine D5 |
| Ibuprofen | - | 205.1234 | 159.1172 | 10 | 13.70 | 14.22 | Gemfibrozil D6 |
| Indomethacin | - | 356.0689 | 297.0560 | 30 | 13.82 | 14.36 | Indomethacin D4 |
| Iodixanol | - | 773.8494 | 126.9042 | 50 | 6.11 | 5.79 | Sulfadiazine D4 |
| Iohexol | + | 821.8868 | 803.8744 | 21 | 3.39 | 2.77 | Acetaminophen D4 |
| Iopromide | + | 791.8755 | 372.9690 | 50 | 4.75 | 4.28 | Acetaminophen D4 |
| Irbesartan | + | 429.2392 | 207.0914 | 25 | 14.53 | 15.14 | Irbesartan D6 |
| Ketoprofen | + | 255.1012 | 105.0334 | 25 | 14.48 | 15.09 | Valsartan D3 |
| Lamotrigine | + | 256.0151 | 172.9667 | 40 | 8.93 | 8.92 | Lamotrigine 13C3 |
| Levofloxacin | + | 362.1511 | 216.1042 | 21 | 7.60 | 7.44 | Ofloxacin D8 |
| Lidocaine | + | 235.1803 | 86.0963 | 18 | 8.16 | 8.07 | Lamotrigine 13C3 |
| Lincomycin | + | 407.2206 | 126.1276 | 24 | 6.22 | 5.91 | Caffeine 13C3 |
| Lorazepam | - | 319.0040 | 283.0274 | 19 | 13.81 | 14.34 | Lorazepam D4 |
| Melamine | + | 127.0727 | 85.0515 | 20 | 1.41 | 0.57 | Cotinine D3 |
| Meprobamate | + | 219.1339 | 158.1175 | 13 | 11.01 | 11.23 | Carisoprodol D7 |
| Metformin | + | 130.1087 | 60.0559 | 15 | 1.83 | 1.03 | Lamotrigine 13C3 |
| Methenamine | + | 141.1135 | 112.0870 | 16 | 1.00 | 0.11 | Cotinine D3 |
| Metoprolol | + | 268.1906 | 116.1068 | 21 | 9.36 | 9.40 | Metoprolol D7 |
| Metronidazole | + | 172.0716 | 128.0453 | 17 | 5.09 | 4.66 | Metronidazole D4 |
| Morphine | + | 286.1436 | 201.0909 | 40 | 3.62 | 3.02 | Gabapentin D10 |
| Nalidixic acid | + | 233.0920 | 205.0606 | 32 | 13.40 | 13.89 | Carbamazepine D10 |
| Naproxen | - | 229.0864 | 158.0373 | 13 | 12.97 | 13.41 | Naproxen D3 |
| Neotame | + | 379.2226 | 172.1330 | 30 | 13.14 | 13.60 | Midazolam 13C6 |
| Oseltamivir | + | 313.2116 | 166.0861 | 18 | 11.74 | 12.04 | Oseltamivir D3 |
| Oxazepam | + | 287.0581 | 241.0524 | 25 | 13.63 | 14.14 | Lorazepam D4 |
| Oxytetracycline | + | 461.1555 | 426.1183 | 25 | 7.40 | 7.22 | Ofloxacin D8 |
| Paroxetine | + | 330.1502 | 192.1183 | 33 | 13.25 | 13.72 | Paroxetine D4 |
| Pentobarbital | - | 225.1242 | 122.0975 | 60 | 13.37 | 13.86 | Irbesartan D6 |
| Phenytoin | - | 251.0821 | 102.0349 | 25 | 13.35 | 13.83 | Irbesartan D6 |
| Pipemidic acid | + | 304.1409 | 217.1084 | 25 | 6.50 | 6.22 | Caffeine 13C3 |
| Pregabalin | + | 160.1337 | 142.1227 | 12 | 5.63 | 5.26 | Sulfadiazine D4 |
| Primidone | + | 219.1128 | 162.0912 | 19 | 8.64 | 8.60 | Acetaminophen D4 |
| Propranolol | + | 260.1642 | 116.1068 | 23 | 12.36 | 12.73 | Propranolol D7 |
| Rosuvastatin | + | 482.1755 | 258.1400 | 43 | 14.10 | 14.67 | Bezafibrate D4 |
| Saccharin | - | 181.9917 | 61.9703 | 36 | 4.00 | 3.44 | Hydrochlorothiazide 13CD2 |
| Salicylic acid | - | 137.0244 | 93.0350 | 16 | 4.80 | 4.33 | Acetaminophen D4 |
| Sitagliptin | + | 408.1253 | 174.0524 | 24 | 10.59 | 10.77 | Fluconazole 13C3 |
| Sotalol | + | 273.1265 | 213.0690 | 24 | 4.57 | 4.08 | Sotalol D6 |
| Sucralose | - | 395.0066 | 59.0134 | 22 | 7.63 | 7.48 | Gemfibrozil D6 |
| Sulfadiazine | + | 251.0596 | 156.0111 | 20 | 5.87 | 5.52 | Sulfadiazine D4 |
| Sulfadimethoxine | + | 311.0801 | 156.0766 | 35 | 12.49 | 12.88 | Sulfamethoxazole 13C6 |
| Sulfaguanidine | + | 214.0524 | 156.0115 | 41 | 2.64 | 1.93 | Valsartan D3 |
| Sulfamerazine | + | 265.0753 | 156.0113 | 21 | 7.25 | 7.06 | Sulfamethazine D4 |
| Sulfamethazine | + | 279.0908 | 204.0436 | 22 | 8.39 | 8.32 | Sulfamethazine D4 |
| Sulfamethizole | + | 271.0319 | 156.0113 | 16 | 8.27 | 8.19 | Sulfamethoxazole 13C6 |
| Sulfamethoxazole | - | 252.0445 | 156.0124 | 15 | 9.54 | 9.60 | Sulfamethoxazole 13C6 |
| Sulfapyridine | + | 250.0644 | 156.0112 | 22 | 6.80 | 6.56 | Sulfamethoxazole 13C6 |
| Sulfathiazole | - | 254.0062 | 156.0124 | 20 | 6.46 | 6.18 | Sulfamethoxazole 13C6 |
| Sulisobenzone (BP4) | - | 307.0279 | 211.0398 | 34 | 9.90 | 10.00 | Sulfamethoxazole 13C6 |
| Tetracycline | + | 445.1605 | 410.1234 | 25 | 8.00 | 7.89 | Sulfamethazine D4 |
| Tramadol | + | 264.1956 | 58.0654 | 15 | 9.49 | 9.54 | Tramadol 13C3D3 |
| Trimethoprim | + | 291.1450 | 230.1161 | 29 | 7.22 | 7.02 | Trimethoprim D9 |
| Valsartan | + | 436.2332 | 207.0915 | 17 | 14.67 | 15.30 | Valsartan D3 |
| Venlafaxine | + | 278.2113 | 58.0654 | 27 | 11.53 | 11.81 | Venlafaxine D6 |
| Warfarin | + | 309.1117 | 163.0388 | 18 | 15.04 | 15.71 | Carbamazepine D10 |
| Zanamivir | + | 333.1405 | 60.2418 | 22 | 1.39 | 0.54 | Cotinine D3 |
| Zonisamide | + | 213.0325 | 150.0549 | 12 | 9.45 | 9.50 | Zonisamide 13C6 |

# Table S5. Validation parameters of the target compounds enriched by the VAE approach at 1, 50 and 200 µg/L (n = 3), sorted by alphabetical order.

| **Compound** | **Low Level 1 µg L^-1^** | | | **Intermediate Level 50 µg L^-1^** | | | **High Level 200 µg L^-1^** | | |
| --- | --- | --- | --- | --- | --- | --- | --- | --- | --- |
|  | **RR (%)** | **RSD (%)** | **ME (%)** | **RR (%)** | **RSD (%)** | **ME (%)** | **RR (%)** | **RSD (%)** | **ME (%)** |
| 1H-Benzotriazole | 93 | 19 | 17 | 100 | 3.0 | 5.3 | 89 | 1.8 | 8.0 |
| 2,2',4,4'-Tetrahydroxybenzophenone (BP2) | 73 | 3.1 | 518 | 138 | 42 | 34 | 89 | 15 | 101 |
| 5-Fluorouracil | 114 | 5.6 | 55 | 100 | 3.3 | 5.3 | 111 | 3.3 | 107 |
| 5-methyl-1-H-benzotriazole | 69 | 8.2 | 323 | 84 | 9.3 | 5.3 | 79 | 7.6 | -13 |
| 6αmethylprednisolone | 37 | 8.0 | 333 | 101 | 4.7 | 7.4 | 107 | 5.0 | 3.9 |
| Acetaminophen | 99 | 4.3 | 1.2 | 103 | 0.6 | 7.8 | 104 | 1.8 | -4.8 |
| Acyclovir | 103 | 6.0 | 68 | 108 | 3.2 | -8.6 | 108 | 3.5 | 156 |
| Amantadine | 59 | 11 | 371 | 85 | 9.1 | 2.9 | 96 | 7.0 | -10 |
| Amoxicillin | 0 | 0.0 | 0 | 0 | 0 | -69 | 0 | 0 | -64 |
| Aspartame | 0 | 0.0 | 0 | 0 | 0 | -92 | 0 | 0 | -92 |
| Atenolol | 54 | 6.1 | 209 | 107 | 4.1 | 0.9 | 106 | 7.2 | 4.1 |
| Atorvastatin | 31 | 40 | 343 | 102 | 4.9 | 10 | 47 | 3.9 | 114 |
| Azithromycin | 0 | 0 | 0 | 118 | 48 | 1637 | 49 | 48 | -47 |
| Bezafibrate | 71 | 7.7 | 205 | 100 | 2.1 | 6.0 | 108 | 1.7 | -5.6 |
| Caffeine | 8.7 | 503 | 79 | 114 | 5.8 | 4.5 | 102 | 1.2 | -0.2 |
| Carazolol | 70 | 13 | 376 | 190 | 55 | 1128 | 92 | 0.6 | 5.2 |
| Carisoprodol | 37 | 5.3 | 416 | 108 | 0.9 | 0.4 | 88 | 1.6 | 14 |
| Chloramphenicol | 127 | 2.3 | 73 | 115 | 53 | 13 | 121 | 26 | 189 |
| Ciprofloxacin | 1085 | 117 | 0 | 104 | 4.4 | 10 | 73 | 3.8 | 26 |
| Citalopram | 47 | 17 | 665 | 100 | 4.3 | 18 | 92 | 4.5 | 49 |
| Clofibric acid | 108 | 4.4 | 86 | 94 | 6.3 | 15 | 122 | 3.2 | 133 |
| Cocaine | 19 | 9.3 | 1270 | 107 | 2.6 | 5.4 | 34 | 2.5 | 208 |
| Codeine | 0 | 0.0 | 0 | 0 | 0 | 0 | 0 | 0 | 0 |
| Cotinine | 43 | 13 | 684 | 104 | 1.4 | 5.5 | 106 | 1.0 | -5.1 |
| Cyclamate | 78 | 11 | 109 | 106 | 1.7 | 3.5 | 110 | 6.0 | 186 |
| Diatrizoic acid | 112 | 1.8 | 189 | 101 | 2.7 | -1.7 | 99 | 4.9 | -0.2 |
| Diclofenac | 0 | 0.0 | 0 | 105 | 0.5 | 4.6 | 138 | 1.5 | -29 |
| Eflornithine | 0 | 0.0 | 0 | 79 | 8.0 | -49 | 142 | 4.2 | -96 |
| Enrofloxacin | 627 | 108 | 93 | 81 | 5.2 | 375 | 78 | 20 | 128 |
| Erythromycin | 17 | 36 | -89 | 73 | 21 | -2.4 | 63 | 42 | -23 |
| Florfenicol | 69 | 12 | 257 | 159 | 11 | -38 | 116 | 3.0 | 212 |
| Fluconazole | 118 | 4.0 | 85 | 87 | 3.3 | -7.0 | 110 | 1.3 | 177 |
| Flumequine | 120 | 72 | 412 | 113 | 3.8 | -4.0 | 102 | 0.8 | 14 |
| Furazolidone | 60 | 6.1 | 238 | 90 | 3.1 | 6.1 | 105 | 7.1 | 2.4 |
| Furosemide | 102 | 3.5 | 73 | 73 | 62 | -43 | 97 | 15 | 141 |
| Gabapentin | 49 | 19 | 409 | 105 | 1.9 | 4.3 | 104 | 1.6 | -7.5 |
| Gemfibrozil | 65 | 8.1 | 225 | 105 | 1.0 | 1.1 | 89 | 2.1 | 244 |
| Hydrochlorothiazide | 100 | 3.9 | 101 | 88 | 6.0 | -1.6 | 103 | 1.4 | 180 |
| Hydroxychloroquine | 2695 | 88 | 0 | 56 | 16 | 104077 | 58 | 34 | 335 |
| Hyoscine | 39 | 9.6 | 3305 | 82 | 11 | -11 | 98 | 18 | 552 |
| Ibuprofen | 89 | 26 | 5557 | 115 | 1.5 | 1.0 | 103 | 5.9 | 124 |
| Indomethacin | 182 | 0.4 | 7.5 | 130 | 8.5 | -20 | 83 | 4.3 | 165 |
| Iodixanol | 181 | 0.9 | 13 | 107 | 5.0 | 2.4 | 112 | 5.1 | 146 |
| Iohexol | 159 | 1.9 | 42 | 99 | 6.6 | 4.5 | 94 | 5.8 | -17 |
| Iopromide | 0 | 0.0 | 0 | 96 | 18 | -1.4 | 104 | 4.4 | -23 |
| Irbesartan | 28 | 51 | 1832 | 107 | 0 | 2.8 | 66 | 1.1 | 51 |
| Ketoprofen | 54 | 17 | 108 | 103 | 1.2 | -0.1 | 100 | 16 | -12 |
| Lamotrigine | 69 | 12 | 230 | 102 | 2.5 | 1.9 | 109 | 1.1 | -6.0 |
| Levofloxacin | 355 | 44 | 5720 | 103 | 3.2 | 0.1 | 74 | 7.9 | 24 |
| Lidocaine | 45 | 5.9 | 182 | 105 | 12 | -76 | 102 | 2.2 | -17 |
| Lincomycin | 51 | 14 | 211 | 92 | 5.6 | 6.5 | 103 | 3.6 | -4.2 |
| Lorazepam | 101 | 8.6 | 82 | 97 | 15 | -0.4 | 95 | 1.5 | 224 |
| Melamine | 112 | 0.1 | 3618 | 116 | 5.5 | 0.0 | 130 | 3.7 | 26 |
| Meprobamate | 0 | 0.0 | 0 | 0 | 0 | 0 | 0 | 0 | 0 |
| Metformin | 63 | 8.1 | 234 | 94 | 11 | 2.6 | 124 | 1.6 | -3.8 |
| Methenamine | 59 | 8.6 | -91 | 112 | 4.8 | 341 | 69 | 3.4 | 2.8 |
| Metoprolol | 53 | 3.5 | 291 | 115 | 3.3 | 8.0 | 111 | 3.1 | -7.3 |
| Metronidazole | 97 | 8.2 | 43 | 102 | 7.3 | 1.4 | 107 | 2.0 | -4.4 |
| Morphine | 0 | 0.0 | 0 | 0 | 0 | 0 | 0 | 0 | 0 |
| Nalidixic Acid | 87 | 47 | 358 | 108 | 1.0 | 0.2 | 111 | 2.4 | 1.7 |
| Naproxen | 201 | 1.1 | -7.1 | 0 | 4.1 | 0.0 | 112 | 8.2 | 84 |
| Neotame | 0 | 0.0 | 188 | 100 | 15.5 | -69 | 25 | 25 | -19 |
| Oseltamivir | 41 | 5.1 | 424 | 105 | 3.8 | 4.5 | 99 | 0.7 | 2.1 |
| Oxazepam | 0 | 0.0 | 0 | 96 | 3.7 | -2.9 | 100 | 2.1 | -1.0 |
| Oxytetracycline | 0 | 0.0 | 0 | 56 | 4.3 | -6.7 | 75 | 18 | -47 |
| Paroxetine | 162 | 1.4 | 23 | 103 | 1.1 | 2.8 | 50 | 25 | -38 |
| Pentobarbital | 0 | 0.0 | 0 | 105 | 5.3 | 3.1 | 106 | 4.9 | 2.9 |
| Phenytoin | 67 | 12 | 346 | 129 | 5.6 | 12 | 115 | 29 | 194 |
| Pipemidic acid | 600 | 102 | 68 | 101 | 5.1 | 136 | 83 | 5.2 | 46 |
| Pregabalin | 178 | 22 | 30 | 103 | 3.2 | 6.3 | 121 | 4.5 | -15 |
| Primidone | 0 | 0.0 | 0 | 90 | 7.7 | 5.3 | 101 | 6.3 | -18 |
| Propranolol | 53 | 6.2 | 296 | 105 | 2.7 | 7.6 | 99 | 3.3 | 1.9 |
| Rosuvastatin | 80 | 5.3 | 160 | 126 | 2.6 | 1.7 | 111 | 2.9 | 0.9 |
| Saccharin | 93 | 5.7 | 279 | 110 | 4.2 | 4.2 | 101 | 2.6 | 183 |
| Salicylic acid | 83 | 3.9 | 347 | 107 | 0.9 | 15 | 108 | 5.7 | 195 |
| Sitagliptin | 68 | 8.4 | 162 | 106 | 3.4 | 1.6 | 104 | 1.4 | -10 |
| Sotalol | 58 | 2.3 | 252 | 105 | 3.2 | 1.8 | 103 | 0.5 | -4.2 |
| Sucralose | 186 | 1.8 | 160 | 91 | 7.0 | 8.4 | 124 | 7.9 | 82 |
| Sulfadiazine | 63 | 1.7 | 223 | 104 | 5.4 | 1.8 | 111 | 1.8 | -7.9 |
| Sulfadimethoxine | 59 | 3.1 | 368 | 101 | 5.8 | 17 | 120 | 0.8 | -6.0 |
| Sulfaguanidine | 0 | 0.0 | 0 | 0 | 0 | 0 | 0 | 0 | 0 |
| Sulfamerazine | 66 | 3.5 | 184 | 107 | 6.4 | 2.0 | 102 | 5.3 | -5.8 |
| Sulfamethazine | 51 | 11 | 295 | 137 | 9.3 | 48 | 109 | 3.2 | -2.3 |
| Sulfamethizole | 43 | 17 | 217 | 102 | 6.6 | 10 | 116 | 6.0 | -8.9 |
| Sulfamethoxazole | 63 | 4.6 | 168 | 107 | 1.5 | 1.8 | 111 | 7.5 | 179 |
| Sulfapyridine | 59 | 7.6 | 308 | 100 | 4.0 | 5.4 | 107 | 12 | -2.2 |
| Sulfathiazole | 75 | 13 | 138 | 111 | 5.2 | 2.2 | 109 | 3.0 | 210 |
| Sulisobenzone (BP4) | 97 | 1.9 | 121 | 91 | 10 | 13 | 114 | 2.1 | 214 |
| Tetracycline | 0 | 0.0 | 0 | 26 | 14 | -31 | 42 | 30 | -25 |
| Tramadol | 30 | 26 | 687 | 109 | 2.8 | 5.6 | 112 | 4.2 | -9.4 |
| Trimethoprim | 55 | 3.9 | 257 | 105 | 2.8 | 4.7 | 119 | 3.3 | -4.2 |
| Valsartan | 82 | 13 | 365 | 105 | 0.9 | 3.3 | 93 | 1.9 | 11 |
| Venlafaxine | 46 | 5.9 | 361 | 103 | 4.9 | 7.3 | 105 | 1.6 | -5.0 |
| Warfarin | 40 | 15 | 536 | 110 | 1.8 | -1.8 | 109 | 3.3 | 5.6 |
| Zanamivir | 0 | 0.0 | 0 | 103 | 2.8 | -3.5 | 92 | 12 | -94 |
| Zonisamide | 0 | 0.0 | 0 | 103 | 2.8 | -3.5 | 118 | 3.1 | -17 |

#

# Table S6. Sample location in coordinates.

| **Sample name** | **Latitude (UTM)** | **Longitude (UTM)** |
| --- | --- | --- |
| SAP2bis | 41.42999 | 2.215175 |
| HBAV | 41.45036842 | 2.19982326 |
| DXSA | 41.44558850 | 2.20261138 |
| RBOP | 41.43877969 | 2.20583093 |
| URB1 | 41.43283058 | 2.20366017 |
| DTA2 | 41.42092226 | 2.21613223 |
| FTAU | 41.41963968 | 2.22703186 |

# Table S7. Limits of detection (LOD) and quantification (LOQ) of the target analytes, sorted by alphabetical order.

| **Compounds** | **LOD (ng L^-1^)** | **LOQ (ng L^-1^)** |
| --- | --- | --- |
| 1H-Benzotriazole | 0.07 | 0.21 |
| 2,2',4,4'-Tetrahydroxybenzophenone (BP2) | 0.16 | 0.50 |
| 5-Fluorouracil | 0.45 | 1.34 |
| 5-methyl-1-H-benzotriazole | 0.12 | 0.37 |
| 6α-methylprednisolone | 0.14 | 0.43 |
| Acetaminophen | 0.16 | 0.49 |
| Acyclovir | 0.16 | 0.48 |
| Amantadine | 0.15 | 0.45 |
| Amoxicillin | 0.00 | 0.00 |
| Aspartame | 0.10 | 0.30 |
| Atenolol | 0.03 | 0.09 |
| Atorvastatin | 0.16 | 0.47 |
| Azithromycin | 0.15 | 0.44 |
| Bezafibrate | 0.13 | 0.40 |
| Caffeine | 0.16 | 0.47 |
| Carazolol | 0.07 | 0.21 |
| Carisoprodol | 0.12 | 0.37 |
| Chloramphenicol | 0.07 | 0.20 |
| Ciprofloxacin | 0.16 | 0.48 |
| Citalopram | 0.14 | 0.41 |
| Clofibric acid | 0.17 | 0.49 |
| Cocaine | 0.12 | 0.36 |
| Codeine | 0.14 | 0.43 |
| Cotinine | 0.09 | 0.26 |
| Cyclamate | 0.09 | 0.27 |
| Diatrizoic acid | 0.31 | 0.92 |
| Diclofenac | 0.10 | 0.29 |
| Eflornithine | 0.44 | 1.20 |
| Enrofloxacin | 0.09 | 0.26 |
| Erythromycin | 0.27 | 0.82 |
| Florfenicol | 0.10 | 0.31 |
| Fluconazole | 0.13 | 0.38 |
| Flumequine | 0.13 | 0.39 |
| Furazolidone | 0.11 | 0.35 |
| Furosemide | 0.10 | 0.29 |
| Gabapentin | 0.09 | 0.28 |
| Gemfibrozil | 0.11 | 0.32 |
| Hydrochlorothiazide | 0.11 | 0.33 |
| Hydroxychloroquine | 0.15 | 0.44 |
| Hyoscine | 0.13 | 0.40 |
| Ibuprofen | 0.26 | 0.77 |
| Indomethacin | 0.06 | 0.19 |
| Iodixanol | 0.11 | 0.32 |
| Iohexol | 0.22 | 0.67 |
| Iopromide | 0.23 | 0.72 |
| Irbesartan | 0.12 | 0.35 |
| Ketoprofen | 0.13 | 0.38 |
| Lamotrigine | 0.11 | 0.33 |
| Levofloxacin | 0.10 | 0.30 |
| Lidocaine | 0.11 | 0.33 |
| Lincomycin | 0.13 | 0.39 |
| Lorazepam | 0.14 | 0.42 |
| Melamine | 0.34 | 1.00 |
| Meprobamate | 0.08 | 0.23 |
| Metformin | 0.29 | 0.86 |
| Methenamine | 0.17 | 0.50 |
| Metoprolol | 0.10 | 0.31 |
| Metronidazole | 0.14 | 0.43 |
| Morphine | 0.11 | 0.33 |
| Nadixilic Acid | 0.14 | 0.42 |
| Naproxen | 0.11 | 0.32 |
| Neotame | 0.14 | 0.42 |
| Oseltamivir | 0.09 | 0.28 |
| Oxazepam | 0.13 | 0.40 |
| Oxytetracycline | 0.12 | 0.36 |
| Paroxetine | 0.15 | 0.44 |
| Pentobarbital | 0.13 | 0.39 |
| Phenytoin | 0.13 | 0.39 |
| Pipemidic acid | 0.19 | 0.58 |
| Pregabalin | 0.16 | 0.49 |
| Primidone | 0.04 | 0.12 |
| Propranolol | 0.15 | 0.46 |
| Rosuvastatin | 0.14 | 0.43 |
| Saccharin | 0.14 | 0.41 |
| Salicylic acid | 0.41 | 1.23 |
| Sitagliptin | 0.16 | 0.49 |
| Sotalol | 0.14 | 0.42 |
| Sucralose | 0.12 | 0.36 |
| Sulfadiazine | 0.12 | 0.36 |
| Sulfadimethoxine | 0.06 | 0.17 |
| Sulfaguanidine | 0.17 | 0.52 |
| Sulfamerazine | 0.09 | 0.26 |
| Sulfamethazine | 0.10 | 0.29 |
| Sulfamethizole | 0.16 | 0.48 |
| Sulfamethoxazole | 0.11 | 0.32 |
| Sulfapyridine | 0.12 | 0.37 |
| Sulfathiazole | 0.13 | 0.40 |
| Sulisobenzone (BP4) | 0.06 | 0.18 |
| Tetracycline | 0.40 | 1.19 |
| Tramadol | 0.11 | 0.33 |
| Trimethoprim | 0.11 | 0.35 |
| Valsartan | 0.10 | 0.29 |
| Venlafaxine | 0.10 | 0.30 |
| Warfarin | 0.15 | 0.45 |
| Zanamivir | 0.22 | 0.65 |
| Zonisamide | 0.28 | 0.84 |

# Table S8. Results of the seven wells samples studied sorted by Log D (pH 7.4). The concentration values shown are in ng L^-1^.

| **Compounds** | **SAP2bis** | **HBAV** | **DXSA** | **RBOP** | **URB1** | **DTA2** | **FTAU** |
| --- | --- | --- | --- | --- | --- | --- | --- |
| 1H-Benzotriazole | 1100 | 326 | 275 | <LOQ | 207 | 124 | 95.1 |
| 2,2',4,4'-Tetrahydroxybenzophenone (BP2) | <LOQ | <LOQ | N/F | N/F | N/F | N/F | N/F |
| 5-Fluorouracil | N/F | N/F | N/F | N/F | N/F | N/F | N/F |
| 5-Methylbenzotriazole | 2122 | 768 | 602 | 5.5 | 427 | 137 | 142 |
| 6α-Methylprednisolone | 86.6 | <LOQ | <LOQ | N/F | N/F | <LOQ | <LOQ |
| Acetaminophen | N/F | N/F | N/F | N/F | N/F | N/F | N/F |
| Acyclovir | N/F | N/F | N/F | N/F | N/F | N/F | N/F |
| Amantadine | 99.0 | 113 | 124 | <LOQ | 15.0 | 6.5 | 5.4 |
| Atenolol | 3.7 | 0.1 | <LOQ | <LOQ | 0.1 | 0.1 | N/F |
| Atorvastatin | N/F | N/F | N/F | N/F | N/F | N/F | N/F |
| Azithromycin | N/F | N/F | N/F | N/F | N/F | N/F | N/F |
| Bezafibrate | 1.6 | N/F | N/F | N/F | 8.7 | <LOQ | N/F |
| Caffeine | <LOQ | <LOQ | <LOQ | <LOQ | <LOQ | <LOQ | <LOQ |
| Carazolol | N/F | N/F | N/F | N/F | N/F | N/F | N/F |
| Carisoprodol | N/F | N/F | N/F | N/F | N/F | N/F | N/F |
| Chloramphenicol | N/F | N/F | N/F | N/F | N/F | N/F | N/F |
| Ciprofloxacin | <LOQ | 5.1 | 2.1 | 0.5 | 7.0 | <LOQ | 0.7 |
| Citalopram | N/F | N/F | N/F | N/F | N/F | N/F | N/F |
| Clofibric acid | N/F | <LOQ | N/F | N/F | <LOQ | <LOQ | N/F |
| Cocaine | <LOQ | <LOQ | <LOQ | <LOQ | <LOQ | N/F | <LOQ |
| Cotinine | 13.8 | <LOQ | 2.0 | <LOQ | 0.2 | <LOQ | <LOQ |
| Cyclamate | N/F | N/F | N/F | N/F | N/F | N/F | N/F |
| Diatrizoic acid | N/F | 0.5 | N/F | 0.9 | N/F | N/F | N/F |
| Diclofenac | 13.5 | 23.2 | 25.6 | N/F | N/F | N/F | N/F |
| Eflornithine | N/F | N/F | N/F | N/F | N/F | N/F | N/F |
| Enrofloxacin | <LOQ | 4.4 | 1.8 | 0.8 | 4.6 | 0.4 | 0.8 |
| Erythromycin | N/F | N/F | <LOQ | N/F | <LOQ | <LOQ | 20.9 |
| Florfenicol | N/F | 6.1 | 1.4 | N/F | N/F | N/F | N/F |
| Fluconazole | 108 | 46.1 | 49.2 | 4.2 | 28.6 | 5.6 | 6.3 |
| Flumenquine | N/F | <LOQ | <LOQ | N/F | N/F | <LOQ | <LOQ |
| Furazolidone | N/F | N/F | N/F | N/F | N/F | N/F | N/F |
| Furosemide | 3.292 | N/F | N/F | N/F | N/F | N/F | N/F |
| Gabapentin | <LOQ | <LOQ | N/F | N/F | N/F | N/F | N/F |
| Gemfibrozil | 3.404 | <LOQ | N/F | N/F | N/F | N/F | N/F |
| Hydrochlorothiazide | 1179 | 104 | 133 | 29.2 | 44.4 | 2.3 | 35.9 |
| Hydroxychloroquine | N/F | N/F | N/F | <LOQ | N/F | N/F | N/F |
| Hyoscine | <LOQ | N/F | N/F | N/F | N/F | N/F | N/F |
| Ibuprofen | N/F | N/F | N/F | <LOQ | N/F | N/F | N/F |
| Indomethacin | N/F | N/F | N/F | N/F | N/F | N/F | N/F |
| Iodixanol | N/F | N/F | N/F | N/F | N/F | N/F | N/F |
| Iohexol | N/F | N/F | 4148 | 576 | N/F | N/F | N/F |
| Iopromide | N/F | N/F | N/F | N/F | N/F | N/F | N/F |
| Irbersartan | 104 | 7.9 | 8.9 | N/F | N/F | N/F | N/F |
| Ketoprofen | 104 | <LOQ | <LOQ | <LOQ | <LOQ | <LOQ | <LOQ |
| Lamotrigine | 784 | 276 | 296 | 23.0 | 146 | 14.1 | 17.6 |
| Levofloxacin | <LOQ | 2.8 | 1.1 | 0.3 | 3.7 | <LOQ | 0.3 |
| Lidocaine | 965 | 56.5 | 61.7 | <LOQ | 12.3 | 15.8 | 16.6 |
| Lincomycin | 36.0 | 88.0 | N/F | N/F | N/F | N/F | N/F |
| Lorazepam | 72.9 | <LOQ | 1.8 | N/F | N/F | N/F | N/F |
| Melamine | <LOQ | 53.1 | 40.8 | 2.3 | 53.7 | 5.6 | 5.2 |
| Metformin | <LOQ | N/F | <LOQ | N/F | N/F | N/F | N/F |
| Methenamine | <LOQ | N/F | N/F | N/F | N/F | N/F | N/F |
| Metoprolol | 109 | 12.2 | 23.1 | N/F | 16.5 | 2.1 | 1.4 |
| Metronidazole | N/F | N/F | N/F | <LOQ | N/F | N/F | N/F |
| Nalidixic acid | 1.3 | N/F | 0.1 | <LOQ | 0.2 | 0.1 | 0.1 |
| Neotame | N/F | N/F | N/F | N/F | N/F | N/F | N/F |
| Oseltamivir | N/F | N/F | N/F | N/F | N/F | N/F | N/F |
| Oxazepam | 58.6 | 1.1 | 1.7 | N/F | N/F | N/F | N/F |
| Oxytetracycline | N/F | N/F | N/F | N/F | N/F | N/F | N/F |
| Paroxetine | N/F | N/F | N/F | N/F | N/F | N/F | N/F |
| Pentobarbital | 18.0 | 1.4 | 2.6 | N/F | 1.4 | 3.8 | 4.7 |
| Phenytoin | 95.8 | 5.6 | 16.7 | 0.8 | 3.7 | 5.3 | 7.1 |
| Pipemidic acid | N/F | 6.9 | 3.1 | 1.2 | 9.8 | 0.5 | 1.3 |
| Pregabalin | <LOQ | <LOQ | <LOQ | N/F | <LOQ | <LOQ | <LOQ |
| Primidone | <LOQ | <LOQ | <LOQ | <LOQ | <LOQ | <LOQ | <LOQ |
| Propranolol | 9.8 | N/F | N/F | N/F | N/F | N/F | N/F |
| Rosuvastatin | N/F | N/F | N/F | N/F | N/F | N/F | N/F |
| Saccharin | 16.7 | N/F | <LOQ | <LOQ | <LOQ | N/F | <LOQ |
| Salicylic acid | N/F | N/F | N/F | N/F | N/F | N/F | N/F |
| Sitagliptin | N/F | N/F | N/F | N/F | N/F | N/F | N/F |
| Sotalol | 24.6 | 2.2 | 4.3 | N/F | N/F | N/F | N/F |
| Sucralose | 34099 | N/F | 9021 | 666 | 3775 | 1342 | 1626 |
| Sulfadiazine | 81.6 | 11.4 | 11.9 | <LOQ | 2.4 | 6.5 | 5.7 |
| Sulfadimethoxine | N/F | 0.7 | N/F | N/F | 0.8 | <LOQ | N/F |
| Sulfamerazine | <LOQ | 0.1 | 0.3 | <LOQ | 0.3 | 2.3 | 1.5 |
| Sulfamethazine | 37.6 | 13.3 | 7.8 | N/F | 4.1 | 5.9 | 4.5 |
| Sulfamethizole | N/F | 0.4 | N/F | 0.2 | 0.4 | 0.7 | 0.4 |
| Sulfamethoxazole | <LOQ | 15.0 | <LOQ | <LOQ | 8.3 | <LOQ | <LOQ |
| Sulfapyridine | 42.3 | 21.0 | 11.0 | 0.7 | 2.7 | 1.3 | 0.5 |
| Sulfathiazole | 99.3 | 3.3 | 2.7 | N/F | N/F | N/F | <LOQ |
| Sulisobenzone (BP4) | N/F | N/F | N/F | N/F | N/F | N/F | N/F |
| Tetracycline | N/F | <LOQ | N/F | N/F | <LOQ | N/F | N/F |
| Tramadol | 30.0 | 49.3 | 28.3 | 0.1 | 4.3 | 1.1 | 0.5 |
| Trimethoprim | N/F | N/F | 1.9 | 0.1 | 0.8 | <LOQ | 0.1 |
| Valsartan | 1266 | 10.4 | 24.4 | N/F | 2.1 | 3.0 | N/F |
| Venlafaxine | <LOQ | 0.1 | <LOQ | <LOQ | 0.1 | <LOQ | <LOQ |
| Warfarin | <LOQ | <LOQ | <LOQ | N/F | 13.5 | <LOQ | <LOQ |
| Zanamivir | N/F | N/F | N/F | N/F | N/F | N/F | N/F |
| Zonisamide | N/F | N/F | N/F | N/F | N/F | N/F | N/F |

# Table S9. Quality Score for each compound in both columns, sorted by log D.

| Compound | QS HSST3 | QS BEH Amide | Log D (pH 7.4) | |
| --- | --- | --- | --- | --- |
| Zanamivir | 5 | 10 | -6,0 |  |
| Oxytetracycline | 0 | 10 | -4,3 |  |
| Pipemidic acid | 10 | 10 | -3,6 |  |
| Cyclamate | 7 | 10 | -3,5 |  |
| Sulisobenzone (BP4) | 10 | 10 | -3,5 |  |
| Iodixanol | 7 | 7 | -3,4 |  |
| Metformin | 2 | 10 | -3,4 |  |
| Tetracycline | 10 | 10 | -3,2 |  |
| Iohexol | 7 | 7 | -3,2 |  |
| Eflornithine | 0 | 10 | -3,0 |  |
| Amoxicillin | 0 | 10 | -2,7 |  |
| Ciprofloxacin | 10 | 10 | -2,2 |  |
| Iopromide | 4 | 7 | -2,1 |  |
| Levofloxacin | 7 | 9 | -2,1 |  |
| Atenolol | 10 | 10 | -1,9 |  |
| Aspartame | 4 | 7 | -1,8 |  |
| Meprobamate | 10 | 7 | -1,8 |  |
| Sotalol | 10 | 10 | -1,6 |  |
| 5-Fluorouracil | 9 | 10 | -1,6 |  |
| Acyclovir | 9 | 4 | -1,5 |  |
| Gabapentin | 10 | 3 | -1,4 |  |
| Pregabalin | 10 | 4 | -1,3 |  |
| Saccharin | 10 | 9 | -1,3 |  |
| Melamine | 5 | 10 | -1,2 |  |
| Trimethoprim | 10 | 10 | -1,2 |  |
| Sulfamethizole | 10 | 6 | -1,1 |  |
| Diatrizoic acid | 7 | 4 | -1,0 |  |
| Phenytoin | 10 | 9 | -1,0 |  |
| Sulfaguanidine | 9 | 10 | -1,0 |  |
| Valsartan | 10 | 4 | -0,9 |  |
| Clofibric acid | 7 | 10 | -0,9 |  |
| Enrofloxacin | 10 | 8 | -0,9 |  |
| Sulfadiazine | 10 | 6 | -0,8 |  |
| Furosemide | 10 | 10 | -0,8 |  |
| Salicylic acid | 10 | 10 | -0,8 |  |
| Flumequine | 10 | 8 | -0,7 |  |
| Primidone | 7 | 3 | -0,7 |  |
| Sulfamethoxazole | 10 | 8 | -0,6 |  |
| Sulfadimethoxine | 10 | 8 | -0,5 |  |
| Lincomycin | 4 | 7 | -0,4 |  |
| Amantadine | 10 | 10 | -0,4 |  |
| Nalidixic acid | 10 | 10 | -0,3 |  |
| Metoprolol | 4 | 10 | -0,3 |  |
| Sucralose | 10 | 4 | -0,2 |  |
| Morphine | 10 | 4 | -0,1 |  |
| Bezafibrate | 10 | 4 | -0,1 |  |
| Hydrochlorothiazide | 10 | 9 | 0,0 |  |
| Sulfathiazole | 10 | 9 | 0,0 |  |
| Sulfamerazine | 10 | 9 | 0,0 |  |
| Metronidazole | 10 | 9 | 0,1 |  |
| Ketoprofen | 7 | 3 | 0,1 |  |
| Cotinine | 10 | 10 | 0,2 |  |
| Sulfamethazine | 10 | 9 | 0,2 |  |
| Caffeine | 10 | 3 | 0,3 |  |
| Codeine | 10 | 7 | 0,3 |  |
| Hyoscine | 10 | 10 | 0,3 |  |
| Warfarin | 10 | 5 | 0,3 |  |
| Acetaminophen | 10 | 3 | 0,4 |  |
| Florfenicol | 10 | 9 | 0,4 |  |
| Sulfapyridine | 10 | 9 | 0,4 |  |
| Furazolidone | 10 | 2 | 0,4 |  |
| Ibuprofen | 10 | 6 | 0,5 |  |
| Naproxen | 7 | 3 | 0,5 |  |
| Zonisamide | 7 | 0 | 0,5 |  |
| Tramadol | 10 | 10 | 0,5 |  |
| Neotame | 10 | 4 | 0,6 |  |
| Oseltamivir | 10 | 7 | 0,6 |  |
| Fluconazole | 10 | 9 | 0,7 |  |
| Indomethacin | 10 | 3 | 0,8 |  |
| Methenamine | 5 | 7 | 1,0 |  |
| Rosuvastatin | 10 | 4 | 1,0 |  |
| Chloramphenicol | 10 | 9 | 1,0 |  |
| Sitagliptin | 10 | 4 | 1,1 |  |
| Carazolol | 10 | 10 | 1,1 |  |
| Propranolol | 10 | 10 | 1,2 |  |
| Cocaine | 10 | 10 | 1,2 |  |
| Irbesartan | 10 | 10 | 1,2 |  |
| Atorvastatin | 10 | 4 | 1,3 |  |
| Lidocaine | 10 | 9 | 1,3 |  |
| Citalopram | 10 | 10 | 1,3 |  |
| 2.2'.4.4'-Tetrahydroxybenzophenone (BP2) | 10 | 9 | 1,4 |  |
| Azithromycin | 10 | 4 | 1,4 |  |
| Diclofenac | 10 | 1 | 1,4 |  |
| Venlafaxine | 10 | 10 | 1,4 |  |
| Paroxetine | 10 | 10 | 1,5 |  |
| 1H-Benzotriazole | 10 | 9 | 1,5 |  |
| Oxazepam | 10 | 6 | 1,5 |  |
| Gemfibrozil | 10 | 8 | 1,6 |  |
| Lamotrigine | 10 | 10 | 1,7 |  |
| 5-Methyl-1H-benzotriazole | 10 | 9 | 1,7 |  |
| Erythromycin | 10 | 4 | 1,7 |  |
| Lorazepam | 10 | 9 | 1,9 |  |
| Pentobarbital | 10 | 8 | 1,9 |  |
| Carisoprodol | 10 | 5 | 1,9 |  |
| Hydroxychloroquine | 10 | 7 | 2,0 |  |
| 6∝-Methylprednisolone | 10 | 6 | 2,0 |  |

**References**

Mechelke, J., Longrée, P., Singer, H., & Hollender, J. (2019). Vacuum-assisted evaporative concentration combined with LC-HRMS/MS for ultra-trace-level screening of organic micropollutants in environmental water samples. *Analytical and Bioanalytical Chemistry*, *411*(12), 2555–2567. https://doi.org/10.1007/S00216-019-01696-3/
